# COVID-19–Associated Orphanhood and Caregiver Death in the United States

**DOI:** 10.1542/peds.2021-053760

**Published:** 2021-12-01

**Authors:** Susan D Hillis, Alexandra Blenkinsop, Andrés Villaveces, Francis B Annor, Leandris Liburd, Greta M Massetti, Zewditu Demissie, James A Mercy, Charles A Nelson, Lucie Cluver, Seth Flaxman, Lorraine Sherr, Christl A Donnelly, Oliver Ratmann, H Juliette T Unwin

**Affiliations:** 1 Centers for Disease Control and Prevention, Atlanta, Georgia.; 2 Department of Mathematics; 3 Department of Pediatrics, Harvard Medical School, Harvard Graduate School of Education, Harvard University and Boston Children's Hospital, Cambridge, Massachusetts; 4 Departments of Social Policy and Intervention; 5 Department of Psychiatry and Mental Health, University of Cape Town, Cape Town, South Africa; 6 Institute for Global Health, University College London, London, United Kingdom; 7 Medical Research Council (MRC) Centre for Global Infectious Disease Analysis, School of Public Health, Imperial College London, London, United Kingdom.; 8 Statistics, University of Oxford, Oxford, United Kingdom.

## Abstract

**BACKGROUND:**

Most coronavirus disease 2019 (COVID-19) deaths occur among adults, not children, and attention has focused on mitigating COVID-19 burden among adults. However, a tragic consequence of adult deaths is that high numbers of children might lose their parents and caregivers to COVID-19–associated deaths.

**METHODS:**

We quantified COVID-19–associated caregiver loss and orphanhood in the United States and for each state using fertility and excess and COVID-19 mortality data. We assessed burden and rates of COVID-19–associated orphanhood and deaths of custodial and coresiding grandparents, overall and by race and ethnicity. We further examined variations in COVID-19–associated orphanhood by race and ethnicity for each state.

**RESULTS:**

We found that from April 1, 2020, through June 30, 2021, >140 000 children in the United States experienced the death of a parent or grandparent caregiver. The risk of such loss was 1.1 to 4.5 times higher among children of racial and ethnic minority groups compared with non-Hispanic White children. The highest burden of COVID-19–associated death of parents and caregivers occurred in Southern border states for Hispanic children, in Southeastern states for Black children, and in states with tribal areas for American Indian and/or Alaska Native populations.

**CONCLUSIONS:**

We found substantial disparities in distributions of COVID-19–associated death of parents and caregivers across racial and ethnic groups. Children losing caregivers to COVID-19 need care and safe, stable, and nurturing families with economic support, quality child care, and evidence-based parenting support programs. There is an urgent need to mount an evidence-based comprehensive response focused on those children at greatest risk in the states most affected.

High coronavirus disease 2019 (COVID-19) mortality rates may have severe unrecognized consequences: large-scale death of parents and caregivers for children.^1,2^ By July 31, 2021, >600,00 people had died of COVID-19 in the United States, with thousands more deaths related directly or indirectly to COVID-19.^3,4^ To date, little attention has focused on children who suffer COVID-19–associated death of parents and coresiding grandparents serving as caregivers and the attendant loss of salient nurturing, financial support, and care.^1,2,5^

The United Nations Children’s Fund (UNICEF) defines orphanhood as death of one or both parents.^6^ The definition includes children losing one parent because they have increased risks of mental health problems, abuse, unstable housing, and household poverty.^7–9^ For children raised by single parents, the COVID-19–associated death of that parent may represent loss of the person primarily responsible for providing love, security, and daily care.^10^ During an unprecedented pandemic, many children are at risk for such loss because 23% of American children live in single-headed households.^11,12^ Children who lose caregivers to the pandemic may face intensified trauma and may have an immediate need for kinship or foster care at a time when pandemic restrictions may limit access to protective services.^12–15^

Beyond parents, grandparents are increasingly indispensable, often providing basic needs. In the United States from 2011 to 2019, 10% of children lived with a grandparent,^16,17^ and in 2019, 4.5 million children lived with a grandparent providing their housing.^16^ Black, Hispanic, and Asian children are twice as likely as White children to live with a grandparent.^18^ The majority of children coresiding with grandparents live with a single parent or no parents.^19^ When custodial grandparents raising grandchildren in the absence of parents die, these children, functionally, face orphanhood a second time. With many caregiving grandparents in highest-risk ages for COVID-19 mortality, children may face a serious new adversity.^20^

COVID-19–associated deaths have disproportionately affected racial and ethnic minority populations and may inequitably affect their children.^21^ Disparities in numbers of children affected might be influenced by variations in fertility, age at childbearing, comorbidities, access to health services, social vulnerability, longevity,^22^ and rates of caregiving by grandparents. Assessing disparities in COVID-19–associated orphanhood and death of grandparent caregivers by race and ethnicity is key to building a response framework that serves children at greatest risk and in greatest need.^23^

We estimated nationally and for each state the numbers of children aged 0 to 17 years who have deceased mothers, fathers, or coresident grandparents because of COVID-19–associated deaths. We developed demographic models to estimate variations by race and ethnicity in numbers and rates of children experiencing COVID-19–associated deaths of caregivers. We then summarized evidence-based recommendations addressing the needs of these children.

## METHODS

### Overview

In this observational modeling study, we used fertility rates, excess mortality and COVID-19 mortality, and household composition data to estimate numbers, rates, and ratios for children aged <18 years affected by COVID-19–associated orphanhood and deaths among caregivers. This approach is similar to methods previously described to quantify global estimates of these outcomes.^24^ We report findings for COVID-19–associated deaths of parents and caregivers for the United States overall and every state individually, disaggregated for Hispanic and non-Hispanic populations, including White, Black, Asian, and American Indian and/or Alaska Native populations (Supplemental Information).

### Data Sources and Populations

We used excess deaths and COVID-19 deaths from the National Center for Health Statistics, from April 1, 2020, to June 30, 2021,^25^ disaggregated by state, age in years (0–14, 15–29, 30–49, 50–64, 65–74, 75–84, and >85), and race and ethnicity.^26^ We use COVID-19–associated deaths to describe the combination of deaths caused directly by COVID-19 or indirectly by associated causes (eg, decreased access to health services), reported as excess deaths. Excess deaths are derived by subtracting quarterly average deaths between 2015 and 2019 from quarterly average deaths in 2020 and 2021. We used the larger value between excess deaths and COVID-19 deaths per age band because we are interested in orphanhood associated with the pandemic.

To estimate numbers of children orphaned by death of a parent, we needed fertility rates of women and men at the same disaggregation level as deaths (categories above) for the years children aged <18 were born (2003–2020). We used birth certificate data from the Centers for Disease Control and Prevention Natality Online Database^27^ to calculate age- and sex-specific fertility rates (average number of children born per woman or man) over time for women aged 15 to 49 and men aged 15 to 77 years (Supplemental Information).

For each state (*a*), sex (*s*), and race and ethnicity (*r*), we calculated the average number of children per adult of a given age, sex, and race and ethnicity, which we further adjusted for childhood mortality and denote by *F*_*a,s,r*_ (Supplemental Information). Data were available for 2003 to 2019 for women and for 2016 to 2019 for men; therefore, we used Poisson regression models for each state, race and ethnicity, and age group to estimate trends in fertility of men (Supplemental Information). Given unavailability of fertility data after 2019, we assumed 2019 rates held constant for 2020 and 2021. Although a recent report showed a 3.7% decline in fertility in late 2020,^28^ this change is unlikely to meaningfully bias our orphanhood estimates for children aged <18 years because it would only affect the early infancy age, and evidence suggests 75% of orphaned children are aged >10 years.^1^

Data on coresiding grandparent caregivers were derived from the 2019 US Census American Community Survey (ACS). ACS measures whether any grandchildren live with a grandparent in the household and whether the grandparent is responsible for their basic needs, including food, shelter, clothing, and day care.^29^ Grandparent responsibility is further classified by absence of the parent in the home (Supplemental Information). We used ACS data routinely tabulated on coresiding grandparents for persons aged ≥30 years^29^ to determine proportions of adults by sex, race and ethnicity, and state who were grandparent caregivers.

### Outcomes

**FIGURE 1:**
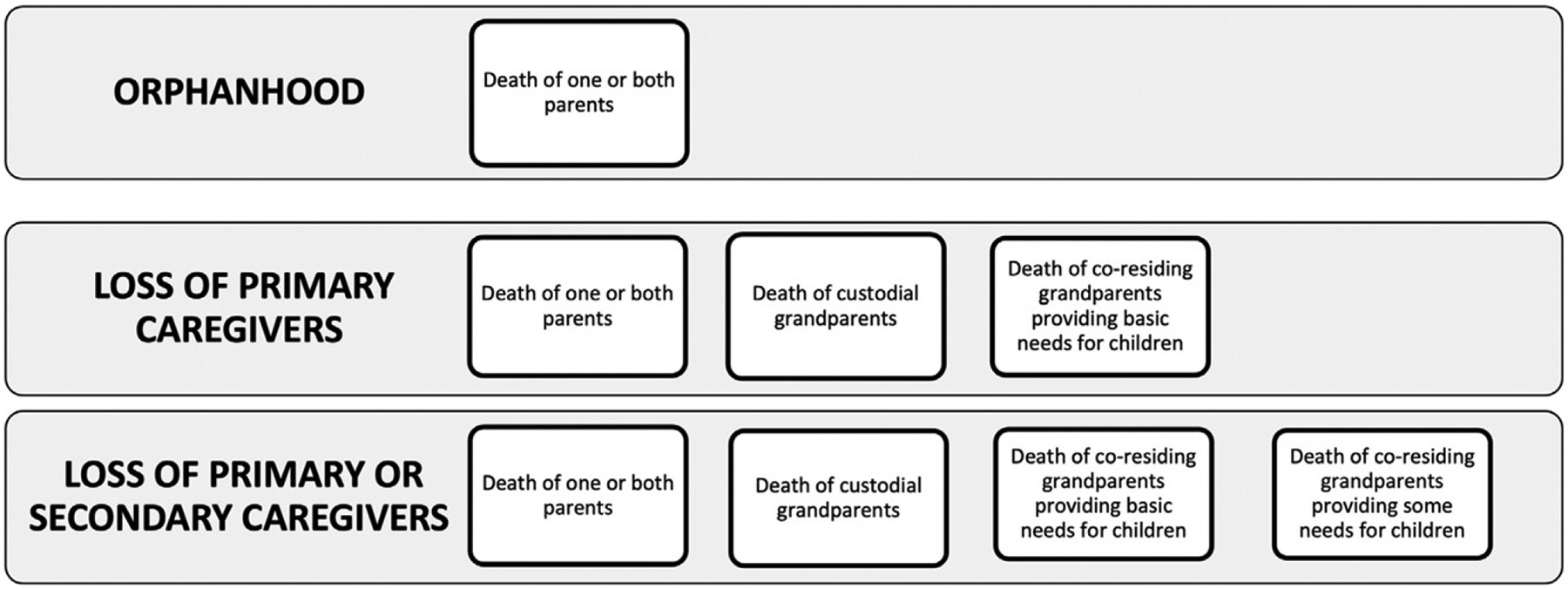
Classification of deaths of parents, custodial grandparents, coresiding grandparents providing most basic needs, and coresiding grandparents providing some basic needs.

Our outcomes were consequences of COVID-19–associated death of parents or coresiding grandparents and included orphanhood, loss of primary caregivers, and loss of primary or secondary caregivers (Fig 1). We defined orphanhood as death of one or both parents.^6^ Primary caregivers have been described as parents or grandparents responsible for most basic needs and care and secondary caregivers as grandparents providing some basic needs or care.^18,30,31^ We defined loss of primary caregivers as the sum of orphanhood, death of custodial grandparents providing parental care for their grandchildren in the absence of parents,^32^ or death of coresiding grandparents (living with grandchild[ren] and child’s parents) responsible for most basic needs (eg, food, housing, clothing, and day care).^18^ We operationalized loss of secondary caregivers as death of grandparents providing housing but not most basic needs.

### Analyses

We estimated numbers of children orphaned by COVID-19–associated death of parents for each state by age category (*a*), sex (*s*), and race and ethnicity (*r*) of parent(s) by multiplying COVID-19 associated deaths in each sex, age, and race and ethnicity bracket (denoted by $D_{a,s,r}^{\text{parent}}$) with the corresponding cohort fertility rates (denoted by *F*_*a,s,r*_):

$$C_{a,s,r}^{\text{orphaned}}=D_{a,s,r}^{\text{parent}}*F_{a,s,r}.$$

To avoid duplicating children who lost both parents, we adjusted estimates on the basis of published household secondary attack rates and infection fatality ratios (Supplemental Information).

To estimate the number of children affected by death of custodial, primary caregiver grandparents, and secondary caregiver grandparents, we multiplied the proportions of the respective grandparent totals derived from the ACS data with COVID-19–associated deaths to produce minimum estimates of custodial, primary, or secondary grandparent caregiver loss (Supplemental Information). To avoid double counting children experiencing deaths of more than one caregiver, we adjusted our estimates as for orphanhood.(Supplemental Information).

We report numbers and rates of children experiencing each outcome, disaggregated by race and ethnicity. We calculated uncertainty for frequency measures from 1000 resampled data sets by summarizing estimates and taking the lower 2.5 and upper 97.5 percentile to obtain 95% bootstrap intervals for central analysis estimates (Supplemental Information). We display state-specific findings using visualizations. Finally, we report rate ratios and 95% bootstrap confidence intervals for variation by race and ethnicity in children’s risk of COVID-19–associated death of caregivers.

## RESULTS

### Burden of COVID-19–Associated Orphanhood and Death of Caregivers

During 15 months of the COVID-19 pandemic, 120 630 children in the United States experienced death of a primary caregiver, including parents and grandparents providing basic needs, because of COVID-19–associated death. Additionally, 22 007 children experienced death of secondary caregivers, for a total of 142 637 children losing primary or secondary caregivers (Table 1). Approximately 91 256 children in racial and ethnic minority groups experienced death of primary caregivers, far surpassing the 51 381 of non-Hispanic White children experiencing such loss (Supplemental Information).

**FIGURE 2:**
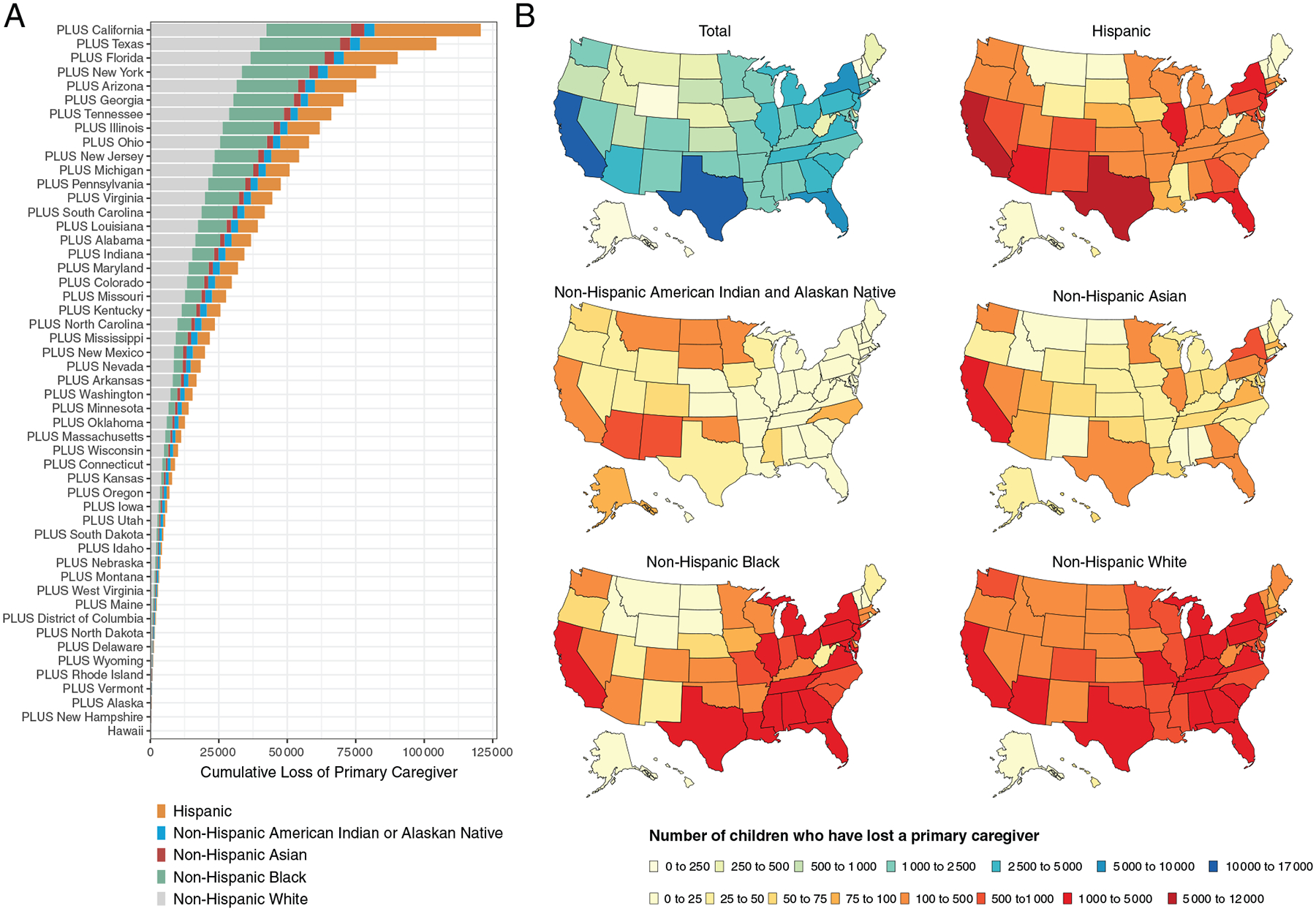
A, Total estimated children losing at least one primary caregiver (parent or custodial grandparent) to COVID-19: cumulative totals across states ordered by number of caregivers lost from bottom lowest (New Hampshire) to top highest (California). B, Estimated children losing at least one primary caregiver by race and ethnicity. PLUS indicates each state is added to the cumulative total shown on the x-axis.

States with large populations had the highest number of children facing COVID-19–associated death of primary caregivers: California (16 179), Texas (14 135), and New York (7175) (Fig 2A). Analyses by race and ethnicity highlight states with disparities in orphanhood and death of primary caregivers (Fig 2B). In southern states, including along the United States–Mexico border, a large proportion of children whose primary caregivers died were of Hispanic ethnicity: 10 863 (67%) in California, 8223 (58%) in Texas, and 773 (49%) in New Mexico. Among children losing caregivers in the Southeast, a large proportion were Black: 1336 (54%) in Louisiana, 1098 (45%) in Alabama, and 1016 (57%) in Mississippi. Affected children who were American Indian and/or Alaska Native were more frequently represented in Arizona 875 (18%), New Mexico 604 (39%), Oklahoma 314 (23%), Montana 175 (38%), and South Dakota 523 (55%).

Variations by race and ethnicity in loss of parent or grandparent caregivers (Table 1) were linked to differences in age composition, family size, family structure, and age-specific mortality rates (Supplemental Figs 2–8). For Hispanic and Black populations, respectively, 7% and 10% of children losing a primary caregiver faced death of grandparents versus 14% among White children.

### Racial and Ethnic Disparities at National and State Levels

**FIGURE 3:**
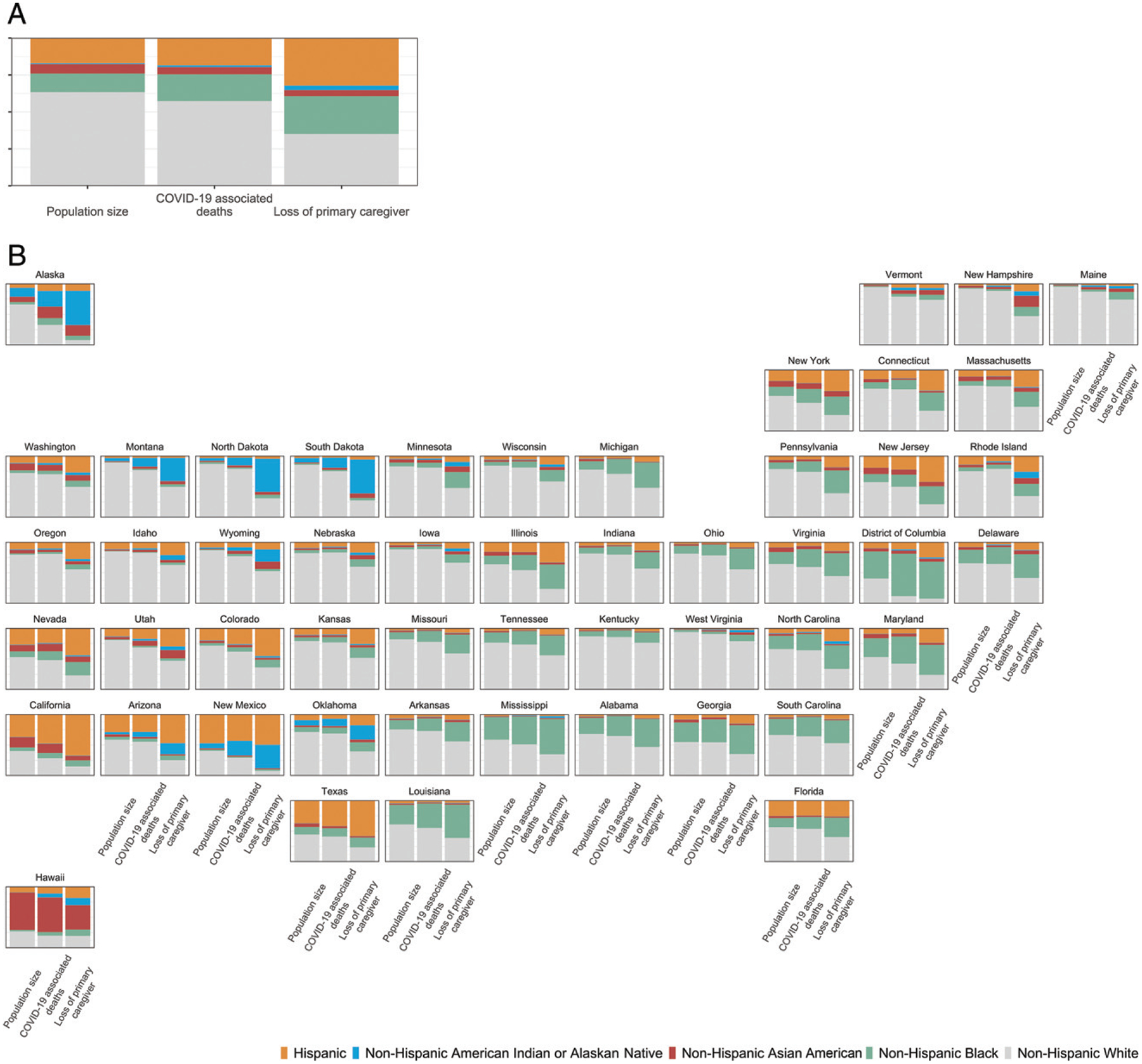
Share of population, COVID-19–associated deaths, and children losing primary caregivers (parents or custodial grandparents) to COVID-19 by race and ethnicity. A, US total. B, By state.

**FIGURE 4:**
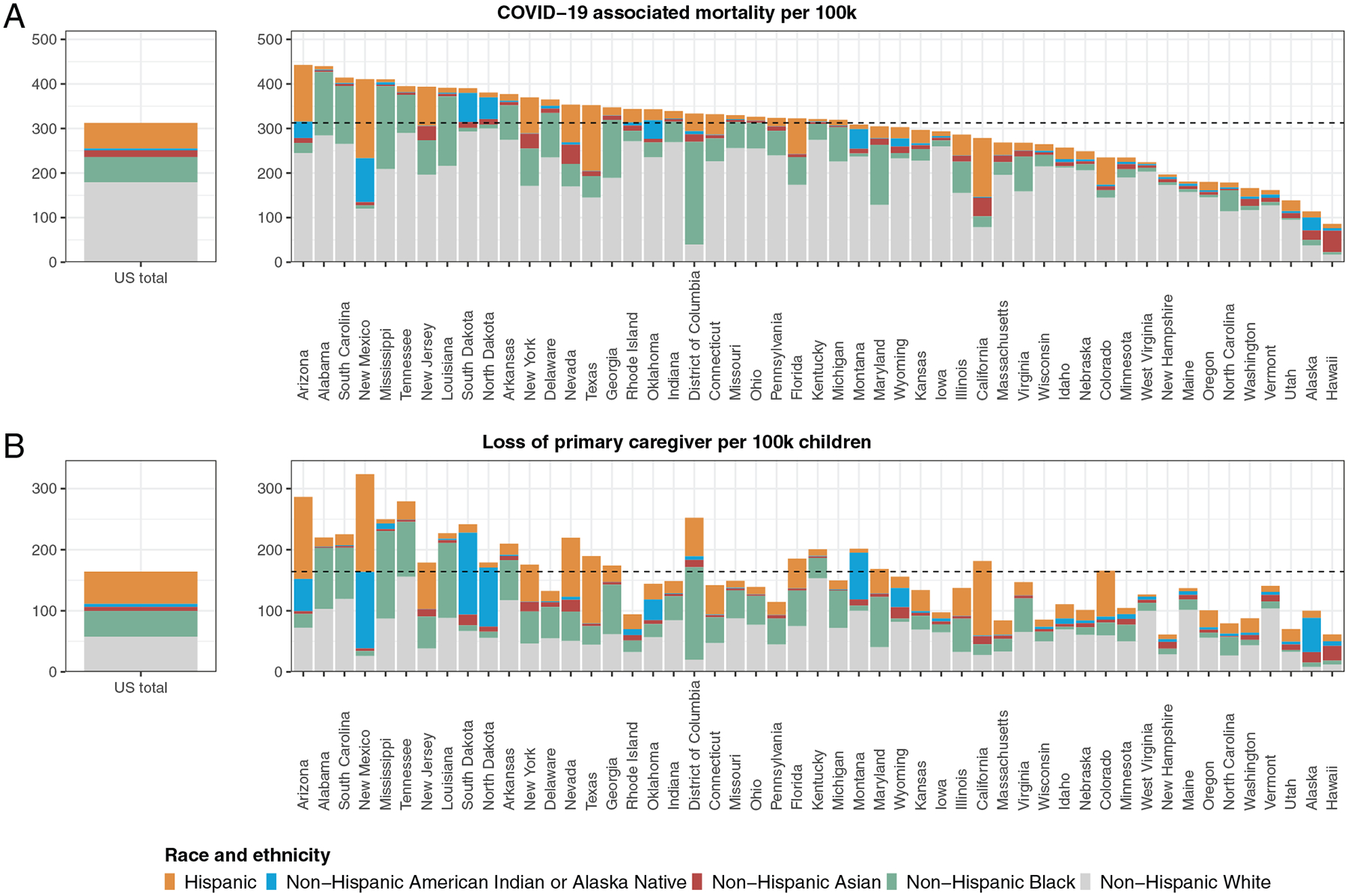
A, COVID-19–associated deaths per state by race and ethnicity per 100 000 residents aged >15 years in each state. B, Estimated loss of primary caregiver per 100 000 children aged <18 in each state by race and ethnicity. Rates at the national level are on the left, and rates at the state level are on the right.

Nationally, COVID-19–associated deaths were distributed across racial and ethnic groups in proportions similar to racial and ethnic distributions of the population (Fig 3A). However, we found substantial disparities in distributions of COVID-19–associated death of primary caregivers across racial and ethnic groups. We estimate White children account for 35% of children who lost primary caregivers, whereas White persons represent 61% of the total population. In contrast, children of racial and ethnic minorities account for 65% of children losing primary caregivers, compared with 39% of the total population. Hispanic and Black children account for 32% and 26%, respectively, of all children losing their primary caregiver compared with 19% and 13% of the total population. Similar patterns occurred across many states (Fig 3B). For example, although Black populations represent <40% of the population and bear 45% and 40% of COVID-19–associated deaths in Mississippi and Louisiana, respectively, Black children comprise the majority of children losing primary caregivers (57% and 54%, respectively). Additionally, we compared COVID-19-associated deaths by race and ethnicity per 100 000 residents aged >15 years (Fig 4A) and estimated loss of primary caregivers per 100 000 children aged <18 years by race and ethnicity (Fig 4B). Both overall and in many states, we found higher mortality rates in White persons and higher rates of loss of primary caregivers among children of racial and ethnic minorities.

Rates of COVID-19–associated death of parents and grandparent caregivers were higher for all racial and ethnic groups than for White children. An estimated 1 of every 753 White children experienced death of their parent or grandparent caregiver compared with 1 of 168 American Indian and/or Alaska Native children (Table 2). Compared with the group at lowest risk, White children, we found American Indian and/or Alaska Native children, Black children, Hispanic children, and Asian children were 4.5, 2.4, 1.8, and 1.1 times more likely, respectively, to lose a parent or caregiver. Consideration of factors that may influence these findings showed death rates and fertility rates were generally higher among non-White than among White persons (Supplemental Table 2).

### States With Highest Disparities for Children in COVID-19–Associated Death of Caregivers

**FIGURE 5:**
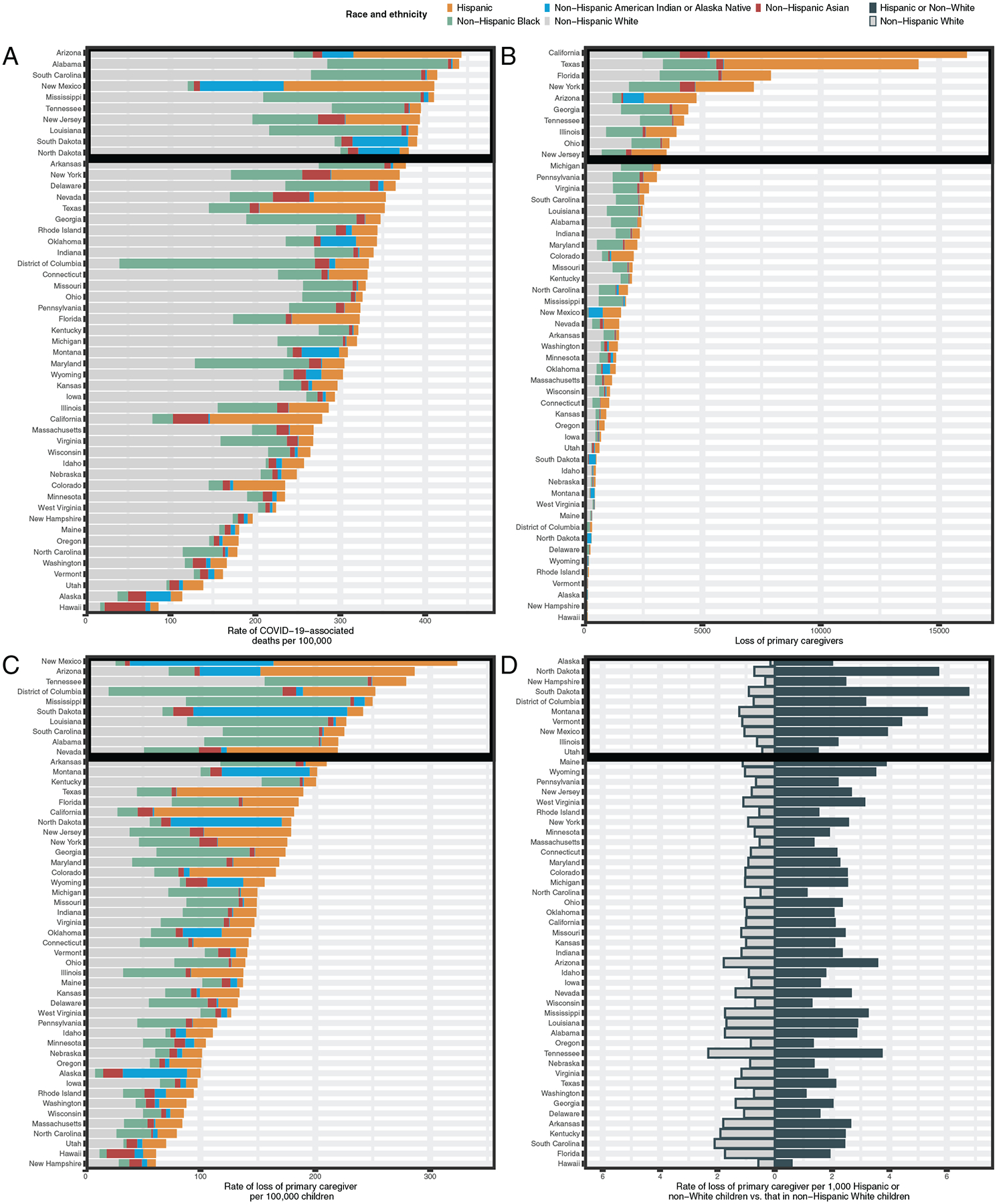
Findings by state and race and ethnicity. A, For reference, rate of COVID-19–associated deaths per 100 000 residents aged >15 years. B, Number of children losing a primary caregiver. C, Rate of loss of primary caregiver per 100 000 children aged 0 to 17 years. D, Rate of loss of primary caregiver per 1000 Hispanic or non-White children and per 1000 non-Hispanic White children (ordered by rate ratio of Hispanic or non-White and/or non-Hispanic children).

We identified states with the greatest racial and ethnic disparities for children affected by death of caregivers to help inform evidence-based responses focused on children at greatest risk in states most affected. First, to provide a reference, we ranked states by COVID-19–associated death rates (Fig 5A). Then, we identified the top 10 states with highest rankings for 3 measures of burden and gap for COVID-19–associated death of primary caregivers: number of children affected, rate of children affected, and comparative rates of children affected among Hispanic or non-White children versus non-Hispanic White children. There was variability between these 3 measures for which states ranked in the top 10 most affected. The highest numbers of children facing death of primary caregivers were observed for California and Texas (25% of total) (Fig 5B), whereas the rate among children of COVID-19–associated deaths of primary caregivers was >1 of 400 children in New Mexico, Arizona, Tennessee, and the District of Columbia (Fig 5C). Among the 5 areas in the US with the largest disparities between non-White and White children in rates of loss of primary caregivers were Alaska, North Dakota, New Hampshire, South Dakota, and the District of Columbia (Fig 5D).

## DISCUSSION

From April 1, 2020, to June 30, 2021, COVID-19–associated deaths accounted for the loss of parents and caregivers for >140 000 children; the lives of these children are permanently changed by the deaths of their mothers, fathers, or grandparents who provided their homes, needs, and care. We observed marked racial and ethnic disparities in the risk of COVID-19–associated orphanhood or death of grandparent caregivers, affecting 1 of 753 White children, 1 of 682 Asian children, 1 of 412 Hispanic children, 1 of 310 Black children, and 1 of 168 American Indian and/or Alaska Native children. The highest burden for children of COVID-19–associated death of primary caregivers occurred in Southern border states for Hispanic children, in Southeastern states for Black children, and in states with tribal areas for American Indian and/or Alaska Native populations. Although less than half of COVID-19–associated deaths occurred in non-White minority populations (4 of 10), the majority of children facing COVID-19–associated orphanhood or deaths of caregivers (almost 7 of 10) occurred in non-White minority populations, with Black, Hispanic, and American Indian and/or Alaska Native children being disproportionately affected.

The COVID-19 pandemic has thrown into sharp contrast the social and health disparities in disease occurrence, severity, and outcomes between geographies and racial and ethnic groups.^33,34^ These disparities impact orphanhood and death of caregivers among children of minority race and ethnicity at much higher rates.^35^ Factors affecting such inequities include structural and social determinants of health, such as discrimination, neighborhood environment, barriers in access to health care, occupation, educational gaps, economic instability, living arrangements, and unstable housing.^36^ These factors increase exposure to severe acute respiratory syndrome coronavirus 2 infection among racial and ethnic minorities because of their disproportionate representation in essential jobs and increased likelihood of living in multigenerational homes. These social determinants of health may have negative impacts on children who face immediate and lifelong consequences of losing a caregiver responsible for their needs and nurture.^35^

Orphanhood and caregiver loss, an adverse childhood experience (ACE), may result in profound long-term impact on health and well-being for children.^37–40^ Loss of parents is associated with mental health problems, shorter schooling, lower self-esteem, sexual risk behaviors, and risks of suicide, violence, sexual abuse, and exploitation.^8,9,39,41–44^ Loss of coresiding grandparents can impact psychosocial, practical, and/or financial support for grandchildren.^5^ After a caregiver’s death, family circumstances may change, and children may face housing instability, separations, and lack of nurturing support.^37,41^ Families with children have been particularly impacted by COVID-19–associated deaths, and Black, Hispanic, and American Indian and/or Alaska Native families have been disproportionally affected.^21^

The death of custodial grandparents may compound the family adversities that led to the child being cared for by grandparents rather than parents. Children living in grandparent-headed, versus parent-headed homes, are more likely to have experienced other ACEs, such as incarceration of a parent, separation or divorce, parental alcohol and/or drug abuse or mental health problems, or domestic violence.^45^ Thus, the death of a grandparent often adds another level of adversity that further increases the likelihood of long-term health and social consequences of ACEs.^36^

Yet there is hope. Safe and effective vaccines can stop COVID-19–associated orphanhood and death of caregivers from negatively impacting children and families. However, formidable challenges persist linked to equitable vaccine access for all racial and ethnic populations and increasing prevalence of variants of concern.^46^

For children who experience COVID-19–associated death of their caregivers, evidence-based responses can help improve short- and long-term outcomes. First, maintaining children in their families, wherever possible, is the priority. This necessitates ensuring families experiencing COVID-19 bereavement are supported and those needing kinship or foster care are rapidly served.^47^ For a strained child welfare system serving >400 000 foster children in 2020, increased investments will be critical, particularly in states with the highest numbers of children affected.^48^

Child resilience after caregiver loss can be bolstered through programs and policies that promote safe, stable, nurturing relationships and address childhood adversity,^35^ including preventing violence and abuse.^49^ Key combination strategies that have strong evidence and established mechanisms of delivery include (1) strengthening economic supports to families, (2) quality child care and educational support, and (3) evidence-based programs to strengthen parenting skills and family relationships.^50,51^

These strategies are critically important when family stressors have led to violence and economic vulnerability; using life course approaches sensitive to the child’s age lessens their risks.^49^ Many community initiatives have innovated the delivery of cost-effective, noncommercial, evidence-based support during COVID-19 restrictions to reach groups most impacted by caregiver loss. Programs need to support family-based alternative care, such as kinship, foster, or adoptive care for children who have no surviving caregivers; such effective programs can prevent violence, reduce substance use, and improve family mental health, with cost-effectiveness of $6 saved for every $1 spent.^52^ These provisions must be sensitive to racial disparities and structural inequalities to reach the children that need them most. The success of these strategies, in the context of disparities, will hinge on engaging community-led initiatives that change the systems driving structural inequities.^36,53^

This study has several limitations. First, the numbers of children experiencing COVID-19–associated orphanhood and caregiver deaths may be underestimated because of delays or underreporting of deaths.^54^ Further underestimation of the numbers of children affected may have occurred because the prevalence for coresiding grandparent caregivers was only available in the ACS for grandparents providing housing. We also considered factors that may have caused our assumption of comparable fertility rates between 2019 and 2020 to bias our estimates of orphanhood. Because excess mortality had the largest absolute impact on men and women who were aged >60 years, we expect any reduction of the denominators for men and women of reproductive age in 2020 to have been minimal, thus not an important source of bias. However, recognizing modest reductions in birth rates in 2020 may have biased our orphanhood estimates, we plan that future estimates will integrate the 2020 and 2021 fertility rates after data becomes available. Additionally, we assumed the race of bereft children matched that of the deceased caregiver, which may have led to overestimates or underestimates of findings by race and ethnicity. Future reports may extend these findings by including sex of deceased caregivers and ages for children affected.

Our analysis extends a previous report from the United States in which highlights increased risk of COVID-19–associated parental bereavement,^1^ by including findings by race and ethnicity. Our article extends the report on global estimates of COVID-19–associated orphanhood in 21 countries, including the Unites States,^2^ by adding state-specific findings by race and ethnicity and using ACS grandparent data. Future pandemic responses will be strengthened by incorporating routine monitoring of children living in the household into vital statistics.^55,56^

## CONCLUSIONS

Our findings suggest an immediate need to integrate care for children into COVID-19 emergency response priorities, which focus on vaccination, mitigation, testing, contact tracing, and disease management. The magnitude of COVID-19–associated parent and caregiver death suggests effective responses should combine equitable access to vaccines with evidence-based programs for bereaved children, focusing on areas with greatest burden and disparities. We propose adding a new pillar of emergency response, “care for children,” to support a comprehensive, 3-pronged approach: prevent, prepare, and protect. The aims of this approach include the following: (1) prevent COVID-19–associated death of caregivers by accelerating equitable access to and uptake of vaccines, (2) prepare safe and loving family-based care support services, and (3) protect children by using evidence-based strategies that address their increased risks of childhood adversity and violence and strengthen their recovery. Because inequalities permeate each of these aims, successful implementation will require intentional investment to address individual, community, and structural inequalities. Effective action to reduce health disparities and protect children from direct and secondary harms from COVID-19 is a public health and moral imperative.^57,58^

## Supplementary Material

Supplementary material

## Figures and Tables

**TABLE 1:** Total Estimated Children Losing Parents and Primary or Secondary Caregiving Grandparents in the United States, by Race and Ethnicity

Race and Ethnicity	Parents	Custodial Grandparents	Coresiding Grandparents Providing Primary Care	Primary Caregiver^a^	Other Coresiding Grandparents	Total^b^
Non-Hispanic White	36 472 (36 146–36 885)	2332 (2321–2339)	3626 (3614–3638)	42 430 (42 094–42 835)	8951 (8924–8979)	51 381 (51 032–51 793)
Non-Hispanic Black	27 831 (27 571–28 172)	1154 (1143–1161)	1883 (1865–1888)	30 868 (30 589–31 218)	3969 (3947–3992)	34 837 (34 547–35 193)
Non-Hispanic American Indian or Alaska Native	3332 (3237–3579)	152 (151–164)	244 (241–254)	3728 (3642–3981)	352 (344–362)	4080 (3990–4330)
Non-Hispanic Asian	4585 (4455–4694)	65 (63–69)	162 (160–168)	4812 (4685–4924)	1331 (1324–1353)	6143 (6013–6269)
Hispanic	36 164 (35 777–36 556)	813 (808–820)	1815 (1803–1825)	38 792 (38 403–39 191)	7404 (7377–7454)	46 196 (45 802–46 631)
Total	108 384 (107 906–109 132)	4516 (4504–4536)	7730 (7705–7750)	120 630 (120 145–121 390)	22 007 (21 969–22 085)	142 637 (142 151–143 482)

a Primary caregivers are parents, custodial grandparents, and coresiding grandparents providing primary care.

b Refers to primary caregivers plus other coresiding grandparents.

**TABLE 2:** Rates of COVID-19–Associated Deaths, Total Fertility Rates, Rates of Loss of Primary or Secondary Caregivers, Proportionate Burden, and Rate Ratio by Race and Ethnicity

Race and Ethnicity	Rates of COVID-19–Associated Death per 100 000 Residents	Total Fertility Rates^a^	Loss of Parents or Caregivers per 100 000 Children Aged <18 y of Each Race and Ethnicity	Proportionate Burden Among Children by Race and Ethnicity	Rate Ratio (Loss of Parents or Caregivers per 100 000 Children/Lost Caregivers per 100 000 Non-Hispanic White Children)
Non-Hispanic White	283 (283–284)	1.77 (1.77–1.77)	133 (132–134)	1 of 753 (746–757)	1
Non-Hispanic Black	448 (446–451)	1.93 (1.93–1.93)	322 (319–325)	1 of 310 (307–313)	2.42 (2.40–2.45)
Non-Hispanic American Indian or Alaskan Native	541 (538–558)	1.99 (1.99–2.00)	592 (579–628)	1 of 168 (159–172)	4.46 (4.35–4.74)
Non-Hispanic Asian	238 (237–241)	1.69 (1.68–1.69)	146 (143–149)	1 of 682 (671–699)	1.10 (1.08–1.13)
Hispanic	339 (338–341)	2.34 (2.34–2.34)	242 (240–245)	1 of 412 (408–416)	1.83 (1.80–1.85)
Total population (without race disaggregation)	313 (312–314)	1.91 (1.91–1.91)	194 (193–195)	1 of 515 (512–518)	—

—, rate ratio not applicable

a Expected number of children aged 0–17 y per woman in 2020.

## WHAT’S KNOWN ON THIS SUBJECT:

Car egiver death increases risks of short-term trauma and lifelong adverse consequences for children. One microsimulation research letter estimated parental deaths in the United States to February 2021. In a global study, researchers aggregated coronavirus disease 2019–associated orphanhood and coresident caregiver deaths across 21 countries.

## WHAT THIS STUDY ADDS:

We report overall for the United States and state–specific findings, disaggregated by race and ethnicity, for coronavirus disease 2019–associated orphanhood and death of grandparent caregivers. High rates of orphanhood, marked disparities, and state-specific differences show the overlooked burden among children at greatest risk in states most affected.

## ABBREVIATIONS

ACE: adverse childhood experience
ACS: American Community Survey
COVID-19: coronavirus disease 2019
