# Supplementary material for: COVID-19–Associated Orphanhood and Caregiver Death in the United States
Source: Pediatrics. Author manuscript; Available in PMC 2024 Feb 26. (PMC10896160; doi:10.1542/peds.2021-053760)
Supplement: Supplementary material [file NIHMS1962864-supplement-Supplementary_material.pdf]

COVID-19-Associated Orphanhood and Caregiver Death in the United States Supplementary Materials

Susan D. Hillis*; Alexandra Blenkinsop;* Andrés Villaveces; Francis Annor; Leandris Liburd; Greta M. Massetti; Zewditu Demissie; Sherr, Lorraine; Christl A. Donnelly; James A. Mercy; Charles A. Nelson, III; Lucie Cluver; Seth Flaxman; Oliver Ratmann**; H. Juliette T. Unwin**

Contents

1 Methods **2**
1.1 Mortality data . . . 2
1.2 Fertility rate . . . 2
1.2.1 Female fertility data . . . 2
1.2.2 Male fertility data . . . 3
1.2.3 Imputation of missing fertility rates . . . 3
1.3 Grandparent data . . . 4
1.4 Numbers of orphans from death of parents . . . 4
1.5 Numbers of children losing care from grandparents . . . 5
1.6 Quantifying Uncertainty . . . 6

2 Results **6**
2.1 Mortality data . . . 6
2.2 Demographics, family size and structures . . . 10
2.2.1 Population size . . . 10
2.2.2 Expected number of children per parent . . . 10
2.2.3 Co-residing grandparents . . . 11
2.3 Deaths of caregivers by state, race and ethnicity . . . 12

1 Methods

Here we describe how we adapted methods previously applied by Hillis et al. to estimate death of caregivers due to COVID-19 using high resolution mortality and fertility data for the United States [1]. Further details on the framework underpinning our approach can be found in work by Lokta et al. [2]

1.1 Mortality data

We extracted total excess deaths and COVID-19 attributable deaths reported to the CDC National Center for Health Statistics (NCHS) by state, age, race and ethnicity from 1 April 2020 through 30th June 2021 [3]. The CDC calculates the excess deaths as the number of deaths recorded in each quarter of 2020-2021 that are above/below the average number of deaths in equivalent quarters from 2015-2019. These are weighted to account for under-reporting of deaths in recent quarters and are presented cumulative over our time period.

Data were available for the following age groupings: 0-14, 15-29, 30-49, 50-64, 65-74, 75-84, 85+ year and race/ethnicities: Hispanic, Non-Hispanic Black, Non-Hispanic White, Non-Hispanic Asian, Non-Hispanic Native Hawaiian or Other Pacific Islander, Non-Hispanic American Indian or Alaska Native and Other. Due to the reporting categories in female fertility data, Non-Hispanic Native Hawaiian or Other Pacific Islanders were grouped together with Non-Hispanic Asian for the analysis.

Strata with fewer than 10 excess deaths were suppressed to meet NCHS confidentially standards, so we imputed missing data at random between 1 and 9. 21.7% of cell counts were suppressed and needed to be imputed, and were most common among younger age groups. For each state, race and ethnicity, we took the larger of excess deaths or COVID-19 attributable deaths for the analysis.

1.2 Fertility rate

The age-specific fertility rate (AFR) per 1000 women or men in state s, age group a and of race and ethnicity r is defined as

$$AFR_{sar} = 1000 \times \frac{\text{Number of live births to women in state } s, \text{ of race/ethnicity } r, \text{ aged } a}{\text{Number of women in state } s, \text{ of race/ethnicity } r, \text{ aged } a}. \tag{1}$$

1.2.1 Female fertility data

We used the 1999-2006 and 2007-2019 natality datasets from CDC WONDER which report on the number of live births from birth certificate data between 2003-2019 [4]. The dataset reported on bridged race categories (4 categories which do not include individuals of more than one race). Specifically, White, Black, Asian (including Native Hawaiian and Other Pacific Islanders), American Indian or Alaska Native. Race and ethnicity were defined as Hispanic (any race) and each of Non-Hispanic White, Black, Asian and American Indian or Alaska Native to match reporting categories of excess death data. Age of mother is reported in 5-year intervals, and we assume a fertility rate of zero for women below 15 and over 50 years of age.

We extracted population data produced by the US Census Bureau in collaboration with the NCHS by year, age and bridged raced categories from the CDC WONDER database.

We summarise below the method taken to obtain fertility rates by year for each state, age of mother, race and ethnicity of mother, and how missing fertility rates were imputed.

- For years 2003-2019:
    1. Extract number of live births of women of a given year, age, bridged race (4 categories), ethnicity.
    2. Extract population sizes for corresponding strata.
    3. Divide (1) by (2) to calculate fertility rates.
- For 2020 assume same fertility rates as 2019.

1.2.2 Male fertility data

The CDC only began collecting data on paternal characteristics since 2016, in the extended natality dataset [5]. Race of father is reported as one of 6 single race categories: White, Black, Asian, American Indian or Alaska Native, Native Hawaiian or Other Pacific Islander, More than one race. Births among Native Hawaiian or Other Pacific Islanders were aggregated with births among Asian fathers to correspond with reporting of female natality data. Age of father is reported in 5-year intervals, and we assume a fertility rate of 0 for men below 15 and over 77 years of age. We chose 77 years as a cut-off since fertility was very low at 60 and this enabled children being born to 60 year olds in 2003 to be included.

Population data were extracted from population estimates produced by the US Census Bureau by year, age and single race categories from the CDC WONDER database [6]. The approach to estimating fertility rates by year for each state, age, race and ethnicity of father is summarised below.

- For years 2016-2019:
  1. Extract number of births of men of a given age, single race (6 categories), ethnicity
  2. Extract population sizes for corresponding strata
  3. Divide (1) by (2) to calculate fertility rates.
- For 2020 assume same fertility rates as 2019.

1.2.3 Imputation of missing fertility rates

To impute missing fertility rates, we used a Poisson model fit to male and female birth counts with a gender and year effect, and population as an offset: 14.8% of female fertility rates were imputed, 18.8% of male fertility rates were imputed between the years natality data was collected (2016-2019) and all male fertility rates were imputed before 2016. We fit to both male and female fertility data, and include gender as a predictor, because historic trends are similar in categories where we have both male and female fertility and this enables us to more accurately estimate the missing data. Our model is fit separately for each state, race/ethnicity and age of parent, and has the form:

$$log(\lambda) = \alpha + \beta_1 X_1 + \beta_2 X_2 \tag{2}$$

where λ is the fertility rate, α is the estimated log rate for men at year 0, β_1 is the estimated effect of year X_1, and β_2 is the estimated effect of being female vs. male, on the log rate. To predict missing fertility rates,

- For women:
  1. The estimated fertility rate for females in year i is $\hat{\lambda}_{if} = \exp(\alpha + \beta_1 X_1 + \beta_2 X_2)$, where $\alpha + \beta_2$ is the estimated log fertility rate for women at year 0 and β_1 is the estimated effect of year $X_1 = i$ on the log rate.
- For men:
  1. The estimated fertility rate for men in year i is $\hat{\lambda}_{im} = \exp(\alpha + \beta_1 X_1)$, where α is the estimated log fertility rate for men at year 0 and β_1 is the estimated effect of year $X_1 = i$ on the log rate.
  2. For strata in which we could not fit a state, race and age-specific model (e.g. due to missing data), models were fit to age and race-specific data across all states, to obtain predictions of fertility rates.
  3. To predict fertility rates in men aged 50+, since female data are not reported for 50+ women models were fit in the same way to male fertility data only, between 2016-2019, across all states.

Figure 1 shows the fertility rates for Hispanic men and women in New York as an example of extracted fertility data, with any missing rates imputed.

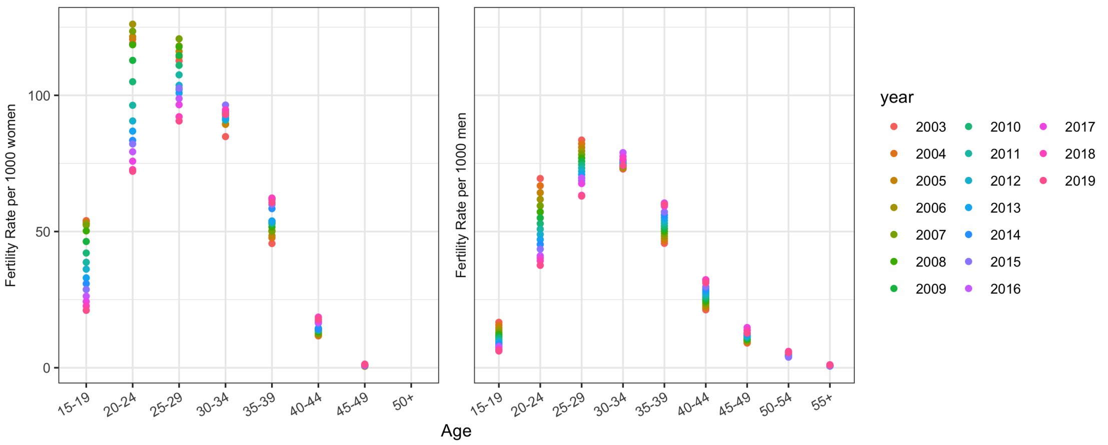

Figure 1: Fertility for men and women of Hispanic origin in New York between 2003-2016. Male data between 2003-2015 estimated using trends over time estimated from Poisson model.

1.3 Grandparent data

Grandparent data were retrieved from the U.S. Census Bureau Table S1002 which summarises total grand-parents living with their own grandchildren under 18 years in each state, collected by the American Community Survey (ACS) [7]. The question asked for respondents is as follows: *Does this person have any of his/her own grandchildren living in this house or apartment?* The survey also summarised the number of grandparents living with grandchildren who are responsible for care, by asking the following question regarding provision of care: *Is this grandparent currently responsible for most of the basic needs of any grandchildren under the age of 18 who live in this house or apartment?* Both totals, which we denote respectively by $G^{30+,\text{co-reside}}$ and $G^{30+,\text{most-responsible}}$, are supplemented for each state with the proportions by race, and the proportion who are reported to be Hispanic, which we combine into the proportion by race/ethnicity, p_r. The totals are also supplemented for each state with the proportions by gender p_s.

Further, the ACS table reports the proportion of grandparents who provide care that live with their grandchild in absence of the parent (custodial grandparents), which we denote by $p_{s,r}^{\text{custodial}}$. Despite there being no direct question in the survey on this, the ACS derive grandparent responsibility from information on the presence of a parent, which is also listed in column "Householder or spouse responsible for grandchildren with no parent of grandchildren present" of Table S1002. We consider grandparents to be primary caregivers to the children if they are responsible custodial grandparents or if the grandparent also lives with a parent of the child but is responsible for the basic needs of the child. Remaining grandparents who live with their grandchildren are classified as secondary caregivers. We estimate the number of grandparents over 30 living with their grandchildren of each sex, race and ethnicity as follows:

$$G_{s,r}^{\text{custodial}} = G^{30+,\text{most-responsible}} \times p_s \times p_r \times p_{s,r}^{\text{custodial}} \tag{3}$$

$$G_{s,r}^{\text{co-reside-primary}} = G^{30+,\text{most-responsible}} \times p_s \times p_r \times \left(1 - p_{s,r}^{\text{custodial}}\right) \tag{4}$$

$$G_{s,r}^{\text{co-reside-secondary}} = G^{30+,\text{co-reside}} \times p_s \times p_r - G_{s,r}^{\text{custodial}} - G_{s,r}^{\text{co-reside-primary}} \tag{5}$$

where $s \in \{\text{male,female}\}$, and as before $r \in$ {Hispanic,Non-Hispanic White, Non-Hispanic Black, Non-Hispanic Asian, Non-Hispanic American Indian or Alaska Native}.

1.4 Numbers of orphans from death of parents

We used methods as described previously [1] to estimate the number of children orphaned and to de-duplicate orphans who may have lost both parents. For each state, we considered the expected number of children of an adult of age a, gender s, and race/ethnicity r in year y from Section 1.2, which we denote by $F_{y,a,s,r}$. We adjusted the expected births for child mortality based on UN estimates of national survival rates to reach

adulthood [8]. Denoting the survival rates of children of age a in year y by $S_{y,a}$, we calcuate the number of children $F_{a,s,r}$ an individual of a given age, sex and race/ethnicity is expected to have in 2020 with

$$F_{a,s,r} = \sum_{i=0}^{17} F_{2020-i,a-i,s,r} \times S_{2020-i,i}, \tag{6}$$

which sums over the yearly category specific expected number of births over the past 18 years while adjusting for childhood mortality, and where a ranges from age 15 to 50 for women, and 15 to 77 for men. We then aggregated the sex-race/ethnicity-specific average number of children in 2020 so they match the age categories in the mortality data (15-29, 30-49, 50-64, 65-74, 75-84).

The age category-sex-race/ethnicity-specific numbers of children orphaned were calculated by multiplying the average number of children per age-sex-race/ethnicity category and the number of COVID-19 associated deaths in this category, $D^{\text{parent}}_{a,s,r}$, according to equation 7:

$$C^{\text{orphaned}}_{a,s,r} = F_{a,s,r} \times D^{\text{parent}}_{a,s,r}, \tag{7}$$

where a corresponds to the age category of the parent (15-29, 30-49, 50-64, 65-74, 75-84), s to the gender of the parent and r to race/ethnicity of the parent.

To obtain the total sex-race specific numbers of children orphaned, we summed over the age groups of the parents, as in equation 8:

$$C^{\text{orphaned}}_{s,r} = \sum_{a \in A} C^{\text{orphaned}}_{a,s,r}, \tag{8}$$

where A=(15-29, 30-49, 50-64, 65-74, 75-84).

We also de-duplicated children who may have lost both parents to COVID-19. For each parent lost, we estimate the number of second parents who were infected and amongst them the number of second parents who died. We conservatively assume that all children have two parents, and we assume that the second parent is of the same age as the first parent. Specifically, we use the household secondary attack rate (SAR) to calculate the number of children whose second parent would get infected and the infection fatality ratio (IFR) to work out how many of those would die [9, 10],

$$C^{\text{double-orphaned}}_{a,s,r} = F_{a,s,r} \times D^{\text{parent}}_{a,s,r} \times \text{IFR}_a \times \text{SAR}, \tag{9}$$

where estimates of household secondary attack rates are taken from [9], and the infection fatality ratios are from [10]. Combining the estimates for both genders and ensuring we are not left with negative counts, we aggregate by gender and race/ethnicity through:

$$C^{\text{double-orphaned}}_{r} = \sum_{a \in \mathcal{A}} \min\left(C^{\text{double-orphaned}}_{a,\text{male},r}, C^{\text{double-orphaned}}_{a,\text{female},r}\right), \tag{10}$$

where $\mathcal{A}$=(15-29, 30-49, 50-64, 65-74, 75-84), s is the gender of the parent, and r is the race/ethnicity of the parent. To de-duplicate orphaned children, we subtract (10) from (8), and list separately de-duplicated orphans of mothers, de-duplicated orphans of father, and double orphaned children.

1.5 Numbers of children losing care from grandparents

To estimate children losing grandparents in one of the three categories of grandparents who co-reside with their grandchildren, we multiplied the number of COVID-19 associated deaths across adults aged 30 years or older by the proportion of adults who are classified as one of the three types of grandparents who provide care, by state, race and ethnicity. Specifically, we calculated

$$C^{\text{loss-custodial-grandparent}}_{s,r} = \frac{G^{\text{custodial}}_{s,r}}{N^{30+}_{s,r}} \times D^{30+}_{s,r} \tag{11}$$

$$C^{\text{loss-primary-grandparent}}_{s,r} = \frac{G^{\text{co-reside-primary}}_{s,r}}{N^{30+}_{s,r}} \times D^{30+}_{s,r} \tag{12}$$

$$C^{\text{loss-secondary-grandparent}}_{s,r} = \frac{G^{\text{co-reside-secondary}}_{s,r}}{N^{30+}_{s,r}} \times D^{30+}_{s,r}, \tag{13}$$

where s corresponds to the gender of the grandparent and r to race/ethnicity of the grandparent, and $N_{s,r}^{30+}$ denotes for each state the number of adults of gender s and race/ethnicity r that are aged 30 or above. We again de-duplicated following the method in equation 10. Throughout, we consider custodial grandparents and those who are responsible for care as primary caregivers, and those who co-reside but did not claim responsibility for childcare are secondary caregivers.

1.6 Quantifying Uncertainty

To quantify uncertainty in estimates, we carried out repeat analyses on bootstrapped samples of data which may carry uncertainty. Namely, we sampled from the excess death data and birth data as follows:

- Mortality data:

    1. Total excess deaths, d_{sgr}, and COVID-19 deaths, c_{sgr}, between Q2 2020 - Q1 2021 were reported for state s, gender $g \in$ {male,female}, race/ethnicity $r \in${Hispanic,Non-Hispanic White, Non-Hispanic Black, Non-Hispanic Asian, Non-Hispanic American Indian or Alaska Native}, age group $a \in \{15-29, 30-49, 50-64, 65-74, 75-84, 85+$. For each strata, we sampled excess deaths and COVID-19 deaths from Poisson distributions, with parameters $\lambda = d_{sgra}$ and $\lambda = c_{sgra}$, respectively.
    2. In instances where excess deaths were negative, we sampled from a Poisson distribution with $\lambda = |d_{sgr}|$, and applied a negative sign to the sampled value.
    3. We then took the maximum value of excess deaths and COVID-19 deaths in each strata.

- Fertility data:

    1. Births b_{sgr} were reported by state s, gender $g \in$ {male,female}, race/ethnicity $r \in${Hispanic,Non-Hispanic White, Non-Hispanic Black, Non-Hispanic Asian, Non-Hispanic American Indian or Alaska Native}, age group $a \in \{15-19, 20-24, 25-29, 30-34, 35-39, 40-45, 45-49, 50-54, 55+\}$ for males, $a \in \{15-19, 20-24, 25-29, 30-34, 35-39, 40-45, 45-49, 50+\}$ for females. We sampled births from a Poisson distribution, with parameter $\lambda = b_{sgr}$.

We carried out all analyses for 1000 resampled data sets. Estimates of caregiver loss were summarised, taking the lower 2.5 and upper 97.5 centile, to obtain 95% bootstrap intervals for central analysis estimates. Denominators for our ratios were not resampled and used as reported from the corresponding sources.

2 Results

2.1 Mortality data

Figure 2 shows the total COVID-19 associated deaths across the study period by age group and gender. The COVID-19 associated deaths are the maximum of total excess deaths and COVID-19 attributable deaths in each strata. The largest burden of absolute mortality is among men aged 65-84, and among women in 75+. Accounting for population structure, Figure 3 indicates the relative burden to be highest among 85+ in both genders. Figures 4 and 5 show the absolute and relative mortality by race and ethnicity. There are clear differences in the structure of deaths by age, with a higher burden among younger non-White and Hispanic men, whilst the burden of absolute deaths is largest among non-Hispanic White men aged 75-84. American Indian and Native Alaskan populations reported the highest mortality among men aged 30-74, and among women aged 65-74. Mortality rates suggest the relative burden is highest among 85+ across all races, suggesting differences in life expectancy may drive the differences in the distribution of deaths by age and race.

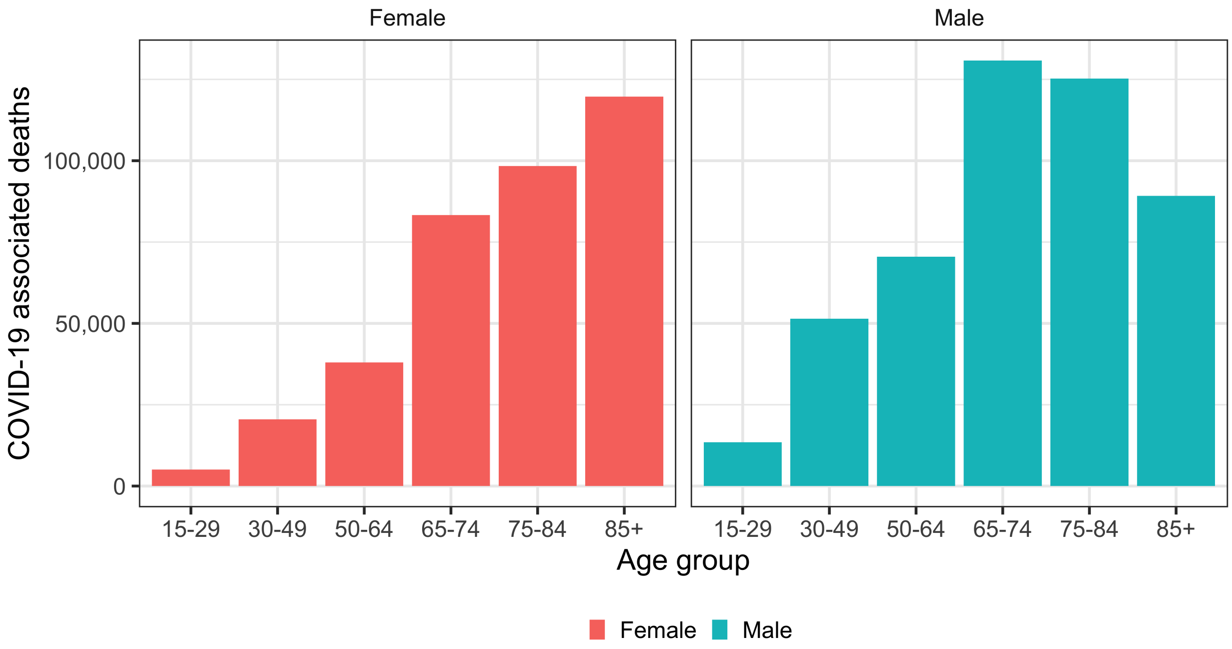

Figure 2: Total COVID-19 associated deaths by age group and gender from January 2020-June 2021.

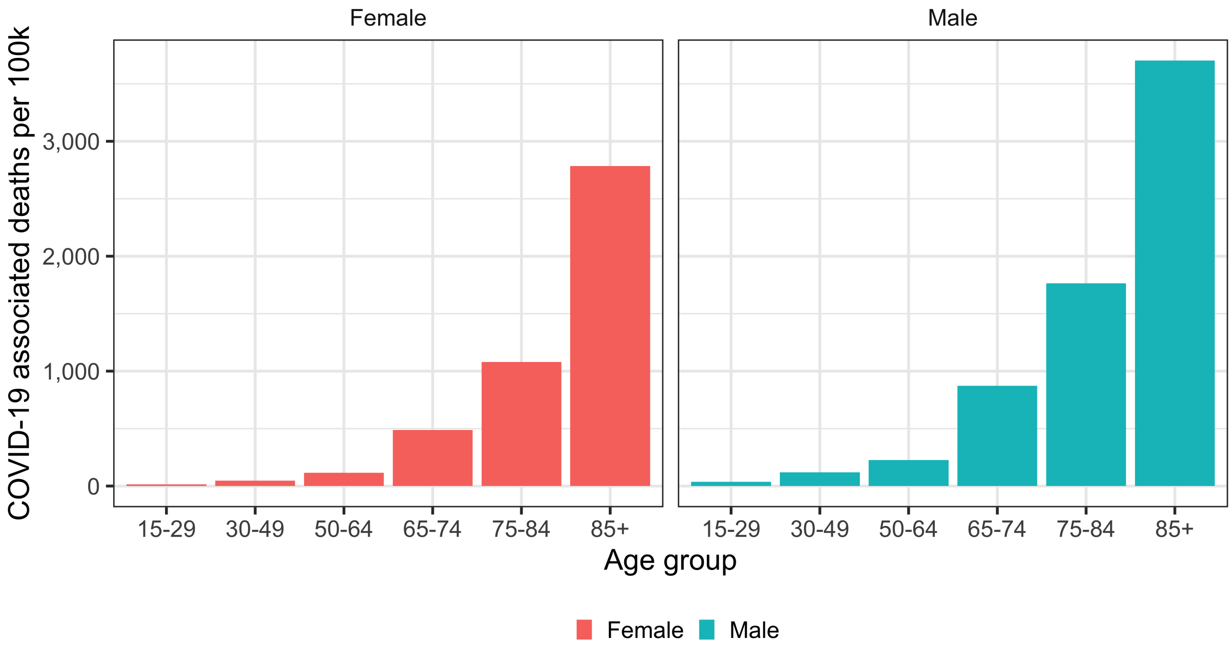

Figure 3: Total COVID-19 associated deaths per 100,000 of each age group and gender from January 2020-June 2021.

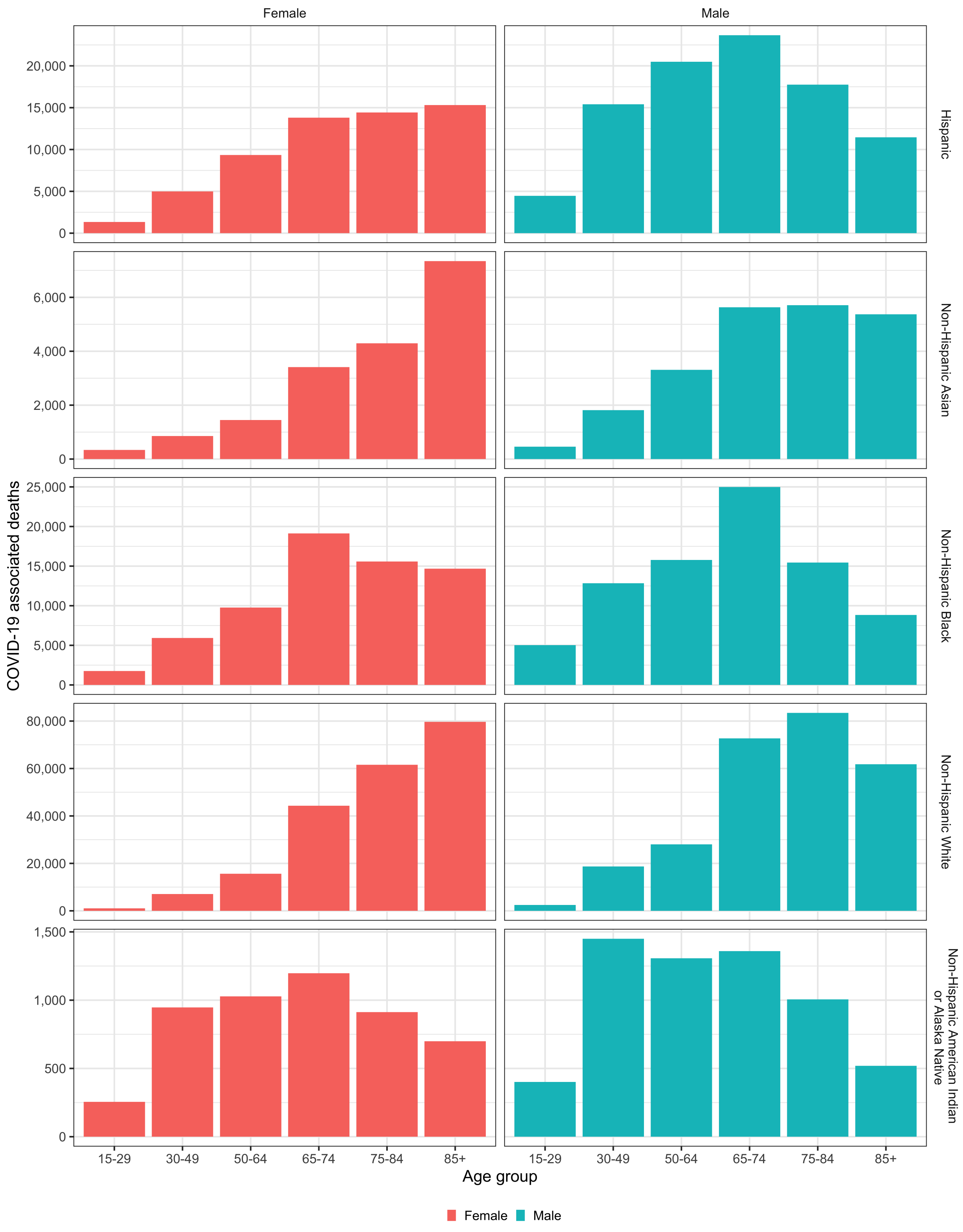

Figure 4: Total COVID-19 associated deaths by age group, gender, race and ethnicity from January 2020-June 2021.

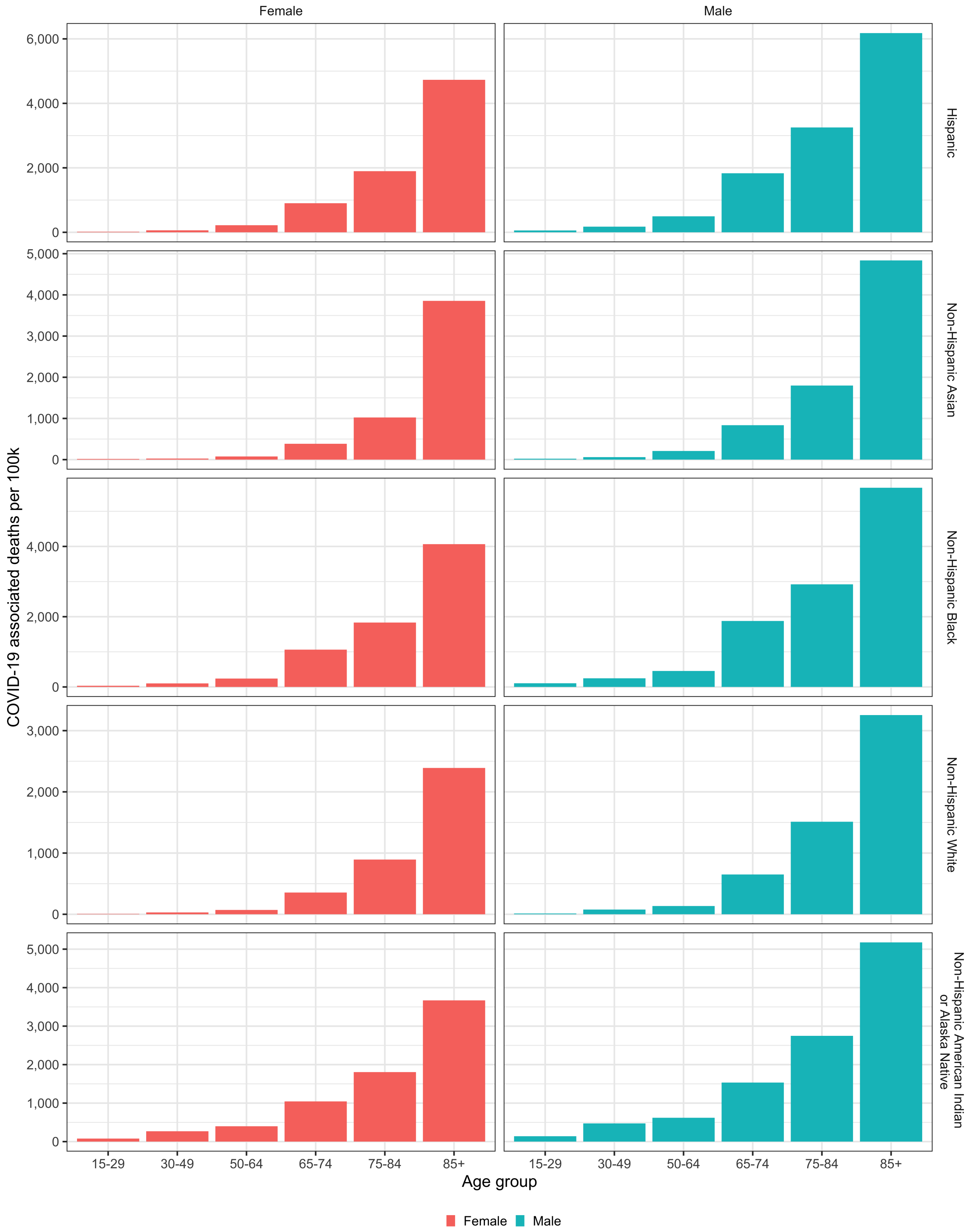

Figure 5: Total COVID-19 associated deaths per 100,000 of each age group, gender, race and ethnicity from January 2020-June 2021.

2.2 Demographics, family size and structures

2.2.1 Population size

Figure 6 shows the population composition by age, race and ethnicity. There are clear differences in population structure; for example the Hispanic population has a very large young population in comparison to non-Hispanic populations.

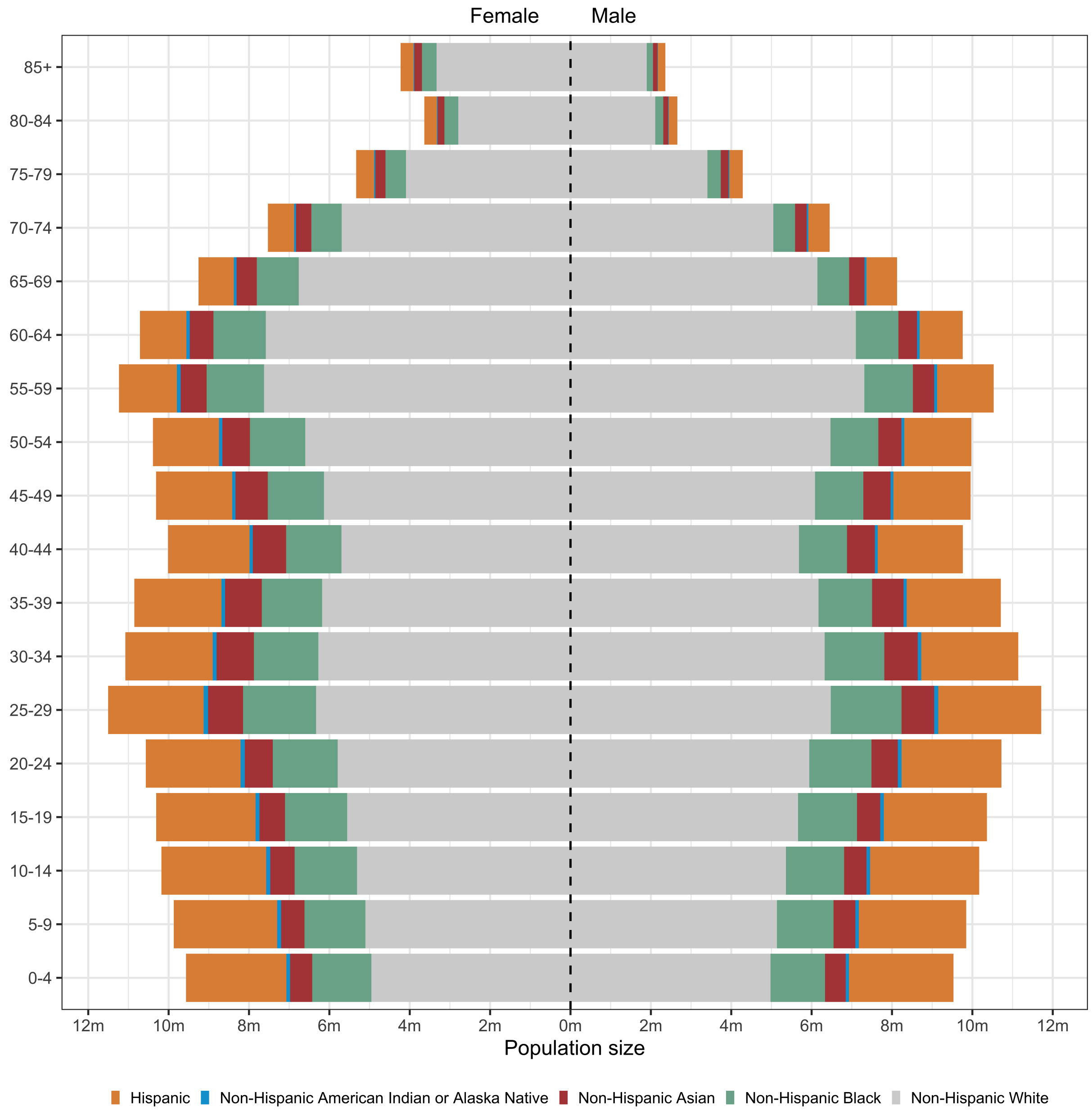

Figure 6: Population size (in millions) by age, race and ethnicity.

2.2.2 Expected number of children per parent

Fertility data from 2003-2019 were used to estimate the expected number of children the average male/female of a given age would have by race and ethnicity in each state. Figure 7 shows the distribution of the expected

number of children for Arizona, as an example. The figure suggests higher fertility rates among the Hispanic population may contribute to more children affected per death of caregiver, compared to deaths among non-Hispanic populations.

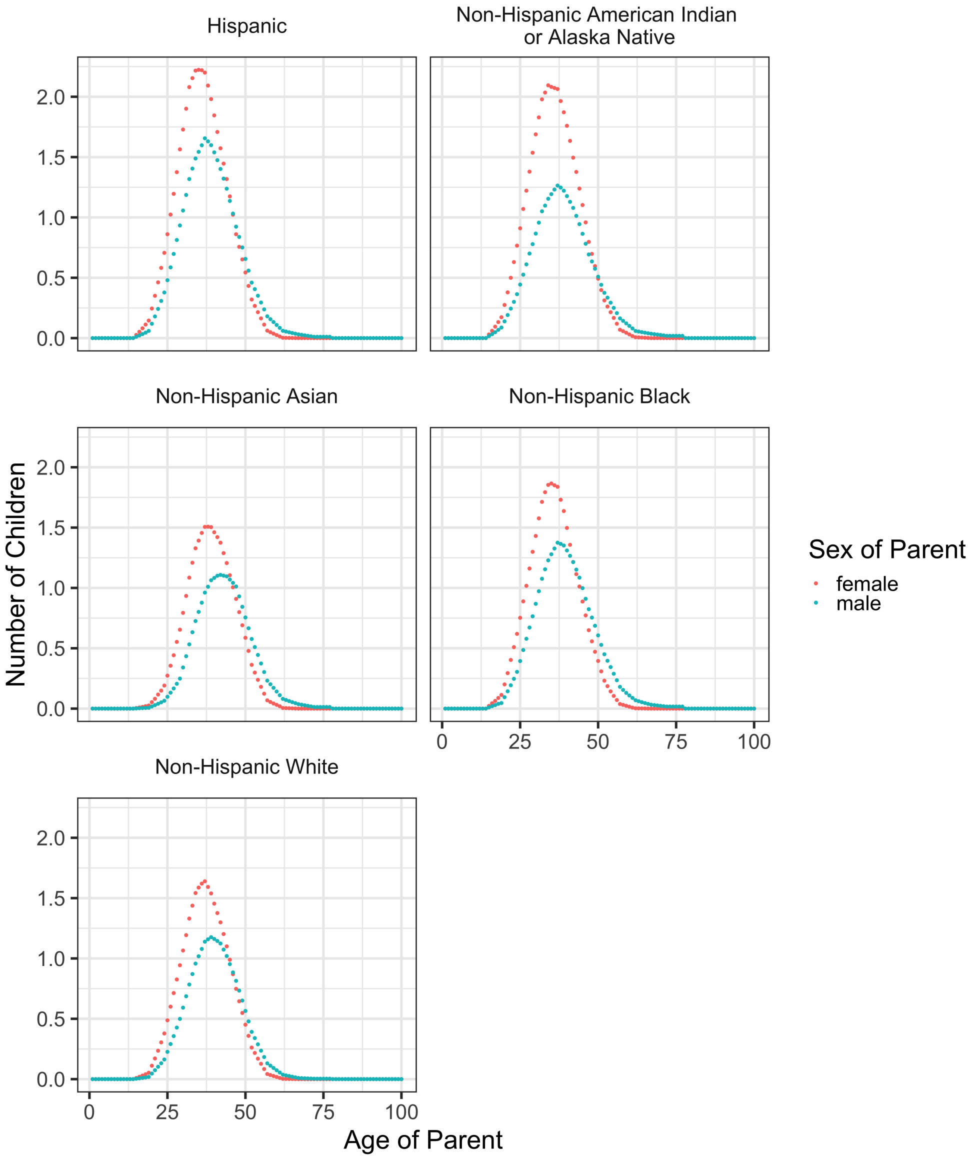

Figure 7: Expected number of children per male/female of a given age in Arizona, by race and ethnicity.

2.2.3 Co-residing grandparents

Figure 8 shows the estimated proportion of the population who are grandparent caregivers who live with their grandchildren, broken down by those who are a) custodial (primary caregivers), b) other co-residing grandparents who live with the children and their parents and are responsible for care (primary caregivers) and c) other co-residing grandparents who provide housing but are not responsible for other basic needs (secondary caregivers). The Hispanic population have the highest proportion of grandparents co-residing with their grandchildren, followed by the American Indian and Alaska Native population, and the non-Hispanic White population have the lowest. Custodial grandparents, who live with their grandchildren in absence of

their parents are most common among the American Indian and Alaska Native population, and approximately half of grandparents who live with their grandchildren are considered to be primary caregivers.

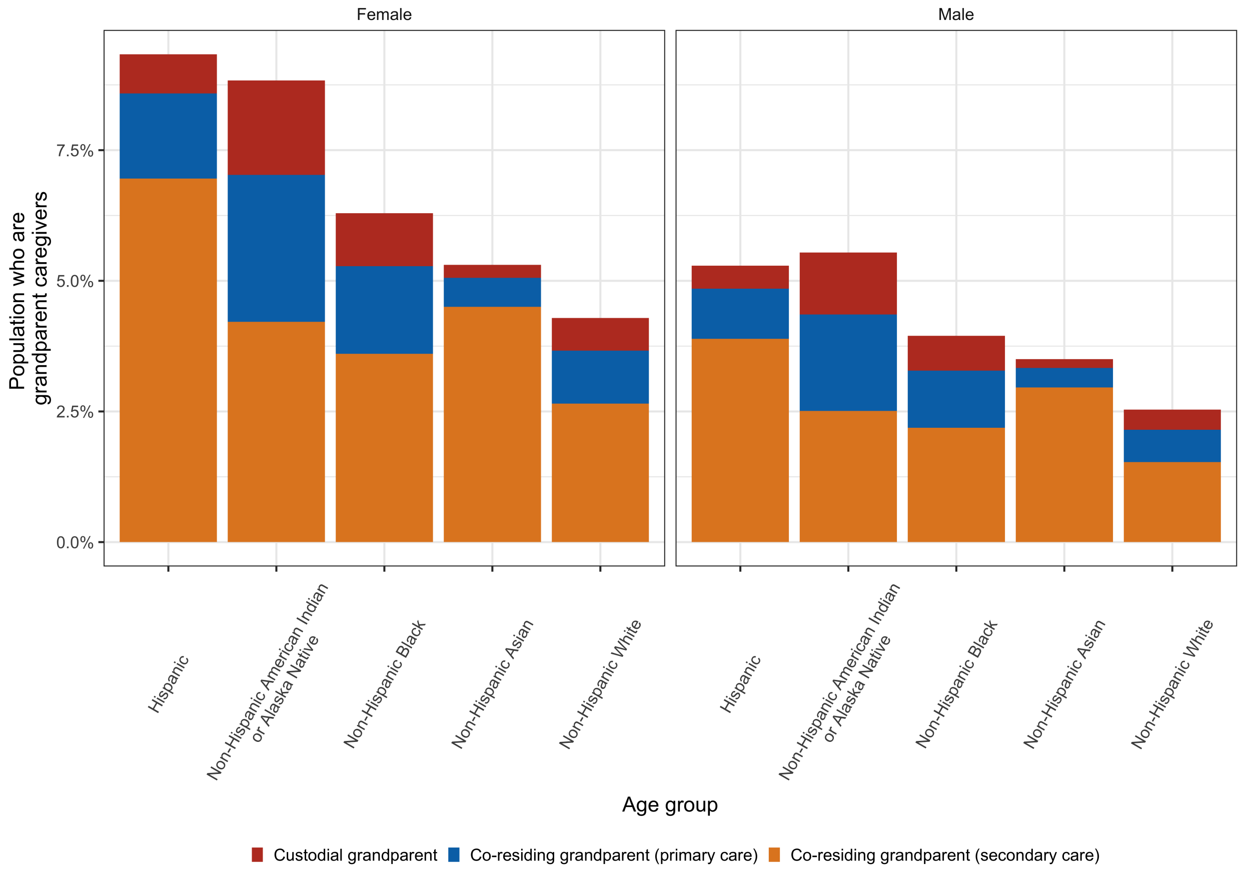

Figure 8: Proportion of adults over the age of 30 who live with their grandchildren, by race and ethnicity.

2.3 Deaths of caregivers by state, race and ethnicity

Table 1 presents the numbers of lost primary caregivers by state, race and ethnicity, ordered by states with the largest total burden. States with large populations (California, Texas, New York and Texas) are estimated to have the highest numbers lost primary caregivers.

Table 2 presents the rates of lost primary caregivers (number of lost caregivers per 100,000 children in each state), ordered by total rates in each state. Smaller states, or rural states with smaller populations, such as the District of Columbia, Wyoming and North Dakota are estimated to have high relative burdens of lost primary caregivers.

Table 1: **Number of children experiencing death of primary caregiver by state and race/ethnicity [95% confidence interval].** Ordered by total in each state. '-' are small counts suppressed due to all data for this category being imputed.

State	Hispanic	Non-Hispanic American Indian or Alaska Native	Non-Hispanic Asian	Non-Hispanic Black	Non-Hispanic White	Total
California	10863 [10665-11078]	107 [93-127]	1168 [1115-1226]	1572 [1500-1639]	2469 [2386-2553]	16179 [15940-16425]
Texas	8223 [8047-8395]	32 [25-42]	296 [268-327]	2251 [2162-2330]	3333 [3237-3426]	14135 [13908-14360]
Florida	2075 [1990-2160]	14 [9-20]	135 [114-152]	2479 [2378-2576]	3196 [3101-3290]	7899 [7741-8060]
New York	2475 [2390-2564]	18 [14-24]	639 [596-683]	2146 [2067-2222]	1897 [1830-1970]	7175 [7034-7318]
Arizona	2230 [2135-2328]	875 [816-947]	76 [62-89]	373 [333-405]	1200 [1146-1261]	4754 [4616-4888]
Georgia	672 [609-733]	11 [8-19]	108 [95-132]	2053 [1969-2132]	1561 [1496-1628]	4405 [4291-4529]
Tennessee	449 [396-497]	9 [9-21]	51 [43-72]	1358 [1287-1427]	2361 [2277-2441]	4228 [4106-4353]
Illinois	1298 [1234-1369]	13 [8-18]	121 [98-134]	1546 [1479-1617]	930 [890-986]	3908 [3800-4026]
Ohio	311 [271-348]	9 [7-19]	59 [41-70]	1225 [1160-1287]	2004 [1927-2072]	3608 [3493-3712]
New Jersey	1479 [1404-1560]	9 [5-16]	233 [210-262]	1020 [965-1077]	747 [711-788]	3488 [3392-3596]
Michigan	314 [281-352]	19 [14-26]	25 [18-40]	1322 [1258-1388]	1557 [1494-1631]	3237 [3142-3347]
Pennsylvania	569 [520-613]	13 [6-20]	149 [134-178]	1138 [1082-1197]	1209 [1158-1255]	3078 [3000-3168]
Virginia	403 [362-446]	8 [5-16]	88 [69-99]	1024 [966-1081]	1221 [1159-1283]	2744 [2652-2838]
South Carolina	202 [173-230]	14 [10-22]	33 [20-37]	943 [891-994]	1345 [1281-1410]	2537 [2443-2624]
Louisiana	99 [78-113]	24 [11-27]	52 [37-66]	1336 [1270-1405]	963 [912-1019]	2474 [2370-2558]
Alabama	163 [130-190]	9 [9-21]	16 [8-24]	1098 [1036-1155]	1137 [1082-1194]	2423 [2332-2512]
Indiana	321 [276-353]	14 [7-17]	59 [41-66]	622 [573-667]	1338 [1275-1397]	2354 [2246-2426]
Maryland	534 [486-589]	9 [6-16]	71 [57-82]	1096 [1041-1153]	542 [502-579]	2252 [2172-2342]
Colorado	953 [886-1019]	63 [51-79]	61 [46-71]	265 [236-300]	758 [713-809]	2100 [2009-2195]
Missouri	154 [127-179]	16 [11-24]	47 [33-55]	626 [578-672]	1206 [1147-1268]	2049 [1964-2119]
Kentucky	108 [85-133]	-	26 [21-41]	334 [304-368]	1546 [1472-1622]	2024 [1943-2116]
North Carolina	392 [354-432]	94 [77-115]	36 [35-56]	706 [664-749]	627 [587-667]	1855 [1790-1936]
Mississippi	49 [40-66]	66 [56-86]	22 [18-35]	1016 [966-1072]	620 [573-664]	1773 [1717-1859]
New Mexico	773 [721-827]	604 [559-659]	21 [15-33]	39 [27-50]	126 [108-145]	1563 [1493-1647]
Nevada	654 [603-705]	33 [26-47]	131 [114-151]	323 [281-356]	344 [315-378]	1485 [1409-1564]
Arkansas	129 [104-152]	13 [8-19]	48 [38-62]	461 [425-498]	825 [778-878]	1476 [1408-1546]
Washington	385 [348-426]	64 [54-82]	127 [109-149]	143 [123-165]	709 [667-754]	1428 [1368-1499]
Minnesota	132 [110-149]	102 [83-129]	123 [105-148]	352 [313-392]	649 [602-696]	1358 [1290-1429]
Oklahoma	239 [207-273]	314 [281-355]	58 [38-60]	199 [174-225]	528 [499-570]	1338 [1268-1404]
Massachusetts	331 [295-362]	17 [7-20]	78 [66-95]	288 [257-316]	470 [440-503]	1184 [1126-1238]
Wisconsin	152 [129-171]	47 [38-67]	55 [45-72]	202 [193-243]	642 [604-681]	1098 [1065-1174]
Connecticut	360 [323-397]	7 [8-34]	27 [21-39]	315 [282-348]	354 [331-386]	1063 [1022-1140]
Kansas	242 [211-278]	20 [16-31]	34 [25-46]	158 [132-186]	486 [448-527]	940 [886-1010]
Oregon	240 [210-271]	33 [24-46]	43 [25-48]	70 [56-88]	482 [449-522]	868 [814-919]
Iowa	74 [60-90]	36 [16-38]	35 [24-45]	96 [77-117]	477 [436-519]	718 [660-761]
Utah	193 [164-227]	41 [33-55]	86 [66-98]	26 [19-41]	306 [267-345]	652 [601-711]
South Dakota	30 [22-44]	290 [252-332]	38 [17-39]	20 [12-34]	145 [126-173]	523 [479-573]
Idaho	105 [86-127]	40 [22-45]	21 [20-43]	19 [11-29]	312 [280-346]	497 [459-544]
Nebraska	83 [68-103]	21 [10-27]	32 [17-38]	59 [46-79]	290 [259-320]	485 [442-521]
Montana	15 [10-26]	175 [148-208]	-	19 [9-24]	229 [202-258]	461 [416-501]
West Virginia	13 [9-21]	-	18 [9-23]	47 [36-63]	363 [333-396]	460 [423-497]
Maine	13 [4-15]	15 [11-27]	19 [14-37]	43 [34-73]	258 [230-289]	348 [328-407]
District of Columbia	85 [71-106]	-	16 [7-19]	204 [186-228]	27 [22-37]	340 [316-375]
North Dakota	15 [14-31]	175 [150-210]	-	18 [14-35]	101 [85-122]	324 [315-392]
Delaware	35 [26-50]	-	16 [7-26]	105 [91-120]	113 [99-131]	272 [249-304]
Wyoming	25 [18-37]	42 [33-59]	-	-	110 [89-122]	209 [180-230]
Rhode Island	52 [36-60]	-	19 [17-31]	40 [29-51]	70 [64-81]	202 [179-225]
Vermont	-	-	-	14 [4-20]	126 [105-146]	171 [146-196]
Alaska	20 [9-27]	95 [72-118]	29 [24-48]	12 [12-28]	14 [10-27]	170 [155-218]
New Hampshire	20 [11-26]	-	29 [14-33]	24 [13-31]	76 [66-91]	161 [128-170]
Hawaii	29 [21-40]	-	62 [43-67]	16 [9-26]	31 [22-43]	157 [128-174]

Table 2: **Number of children experiencing death of primary caregiver per 100,000 children per state [95% confidence interval].** Ordered by total rates in each state. '-' are small counts suppressed due to all data for this category being imputed.

State	Hispanic	Non-Hispanic American Indian or Alaska Native	Non-Hispanic Asian	Non-Hispanic Black	Non-Hispanic White	Total
Alabama	15 [12-17]	1 [1-2]	1 [1-2]	100 [94-105]	103 [98-108]	220 [212-228]
Alaska	12 [5-16]	56 [42-69]	17 [14-28]	7 [7-16]	8 [6-16]	100 [91-128]
Arizona	134 [129-140]	53 [49-57]	5 [4-5]	22 [20-24]	72 [69-76]	287 [278-295]
Arkansas	18 [15-22]	2 [1-3]	7 [5-9]	66 [60-71]	117 [111-125]	210 [200-220]
California	122 [120-124]	1 [1-1]	13 [13-14]	18 [17-18]	28 [27-29]	182 [179-184]
Colorado	75 [70-80]	5 [4-6]	5 [4-6]	21 [19-24]	60 [56-64]	166 [159-173]
Connecticut	48 [43-53]	1 [1-5]	4 [3-5]	42 [38-46]	47 [44-52]	142 [136-152]
Delaware	17 [13-24]	-	8 [3-13]	51 [44-58]	55 [48-64]	133 [121-148]
District of Columbia	63 [53-79]	-	12 [5-14]	151 [138-169]	20 [16-27]	252 [235-278]
Florida	49 [47-51]	0 [0-0]	3 [3-4]	58 [56-60]	75 [73-77]	185 [182-189]
Georgia	27 [24-29]	0 [0-1]	4 [4-5]	81 [78-84]	62 [59-64]	174 [170-179]
Hawaii	11 [8-16]	-	24 [17-26]	6 [4-10]	12 [9-17]	61 [50-68]
Idaho	23 [19-28]	9 [5-10]	5 [4-10]	4 [2-6]	70 [62-77]	111 [102-121]
Illinois	46 [43-48]	0 [0-1]	4 [3-5]	54 [52-57]	33 [31-35]	137 [134-142]
Indiana	20 [17-22]	1 [0-1]	4 [3-4]	39 [36-42]	85 [81-88]	149 [142-153]
Iowa	10 [8-12]	5 [2-5]	5 [3-6]	13 [10-16]	65 [59-70]	97 [90-103]
Kansas	35 [30-40]	3 [2-4]	5 [4-7]	23 [19-27]	69 [64-75]	134 [126-144]
Kentucky	11 [8-13]	-	3 [2-4]	33 [30-36]	153 [146-161]	201 [193-210]
Louisiana	9 [7-10]	2 [1-2]	5 [3-6]	123 [117-129]	88 [84-94]	227 [217-235]
Maine	5 [2-6]	6 [4-11]	7 [6-15]	17 [13-29]	102 [91-114]	137 [129-160]
Maryland	40 [36-44]	1 [0-1]	5 [4-6]	82 [78-86]	41 [38-43]	169 [163-175]
Massachusetts	24 [21-26]	1 [0-1]	6 [5-7]	20 [18-22]	33 [31-36]	84 [80-88]
Michigan	15 [13-16]	1 [1-1]	1 [1-2]	61 [58-64]	72 [69-75]	150 [145-155]
Minnesota	10 [8-11]	8 [6-10]	9 [8-11]	27 [24-30]	50 [46-54]	105 [99-110]
Mississippi	7 [6-9]	9 [8-12]	3 [3-5]	143 [136-151]	87 [81-94]	250 [242-262]
Missouri	11 [9-13]	1 [1-2]	3 [2-4]	46 [42-49]	88 [83-92]	149 [143-154]
Montana	7 [4-11]	77 [65-91]	-	8 [4-10]	100 [88-113]	202 [182-219]
Nebraska	17 [14-22]	4 [2-6]	7 [4-8]	12 [10-17]	61 [54-67]	101 [92-109]
Nevada	97 [89-104]	5 [4-7]	19 [17-22]	48 [42-53]	51 [47-56]	220 [208-231]
New Hampshire	8 [4-10]	-	11 [5-13]	9 [5-12]	29 [25-35]	61 [49-65]
New Jersey	76 [72-80]	0 [0-1]	12 [11-13]	52 [50-55]	38 [36-40]	179 [174-185]
New Mexico	160 [149-171]	125 [116-136]	4 [3-7]	8 [6-10]	26 [22-30]	324 [309-341]
New York	61 [59-63]	0 [0-1]	16 [15-17]	53 [51-54]	46 [45-48]	176 [172-179]
North Carolina	17 [15-18]	4 [3-5]	2 [1-2]	30 [28-32]	27 [25-29]	79 [77-83]
North Dakota	8 [8-17]	97 [83-116]	-	10 [8-19]	56 [47-67]	179 [174-217]
Ohio	12 [10-13]	0 [0-1]	2 [2-3]	47 [45-50]	77 [74-80]	139 [135-143]
Oklahoma	26 [22-29]	34 [30-38]	6 [4-6]	21 [19-24]	57 [54-61]	144 [137-151]
Oregon	28 [24-31]	4 [3-5]	5 [3-6]	8 [7-10]	56 [52-61]	101 [95-107]
Pennsylvania	21 [19-23]	0 [0-1]	6 [5-7]	42 [40-45]	45 [43-47]	115 [112-118]
Rhode Island	24 [17-28]	-	9 [8-14]	19 [14-24]	33 [30-38]	94 [83-105]
South Carolina	18 [15-20]	1 [1-2]	3 [2-3]	84 [79-88]	119 [114-125]	225 [217-233]
South Dakota	14 [10-20]	134 [116-153]	18 [8-18]	9 [6-16]	67 [58-80]	242 [221-265]
Tennessee	30 [26-33]	1 [1-1]	3 [3-5]	90 [85-94]	156 [150-161]	279 [271-287]
Texas	110 [108-113]	0 [0-1]	4 [4-4]	30 [29-31]	45 [43-46]	190 [187-193]
Utah	21 [18-24]	4 [4-6]	9 [7-11]	3 [2-4]	33 [29-37]	70 [65-77]
Vermont	-	-	-	12 [3-16]	104 [86-120]	141 [120-161]
Virginia	22 [19-24]	0 [0-1]	5 [4-5]	55 [52-58]	65 [62-69]	147 [142-152]
Washington	24 [21-26]	4 [3-5]	8 [7-9]	9 [8-10]	44 [41-46]	88 [84-92]
West Virginia	4 [2-6]	-	5 [2-6]	13 [10-17]	100 [92-109]	127 [117-137]
Wisconsin	12 [10-13]	4 [3-5]	4 [4-6]	16 [15-19]	50 [47-53]	86 [83-91]
Wyoming	19 [13-28]	31 [25-44]	-	-	82 [66-91]	156 [134-172]

Table 3: **Total estimated children losing parents, and primary or secondary caregiving grandparents, in the US, by state [95% confidence interval].** Ordered by total in each state.

* Primary caregivers are parents, custodial grandparents and co-residing grandparents providing primary care.
† Refers to primary caregivers plus other co-residing grandparents

State	Parents (a)	Custodial grandparents (b)	Co-residing grandparents providing primary care (c)	Primary caregiver* (a+b+c)	Other co-residing grandparents (d)	Total† (a+b+c+d)
California	15144 [14910-15390]	246 [244-249]	789 [783-795]	16179 [15940-16425]	3703 [3676-3730]	19882 [19634-20151]
Texas	12613 [12391-12835]	509 [505-513]	1013 [1004-1020]	14135 [13908-14360]	2642 [2621-2661]	16777 [16539-17016]
Florida	7199 [7042-7357]	250 [248-253]	450 [445-454]	7899 [7741-8060]	1580 [1565-1594]	9479 [9314-9641]
New York	6549 [6410-6690]	173 [170-175]	453 [449-458]	7175 [7034-7318]	1678 [1665-1695]	8853 [8710-9008]
Arizona	4309 [4174-4442]	137 [135-140]	308 [303-312]	4754 [4616-4888]	733 [723-744]	5487 [5342-5631]
Georgia	3859 [3744-3978]	213 [211-217]	333 [330-339]	4405 [4291-4529]	816 [806-827]	5221 [5105-5354]
Tennessee	3773 [3654-3895]	208 [205-212]	247 [244-252]	4228 [4106-4353]	482 [476-490]	4710 [4584-4842]
Illinois	3566 [3458-3681]	110 [108-112]	232 [230-236]	3908 [3800-4026]	721 [713-734]	4629 [4519-4752]
New Jersey	3225 [3129-3334]	65 [64-67]	198 [194-200]	3488 [3392-3596]	806 [796-816]	4294 [4194-4403]
Ohio	3138 [3028-3239]	213 [210-216]	257 [254-261]	3608 [3493-3712]	603 [596-610]	4211 [4095-4317]
Pennsylvania	2660 [2584-2749]	153 [151-155]	265 [260-267]	3078 [3000-3168]	743 [735-753]	3821 [3741-3916]
Michigan	2924 [2829-3032]	113 [112-115]	200 [198-204]	3237 [3142-3347]	549 [540-555]	3786 [3684-3898]
Virginia	2464 [2375-2560]	109 [106-111]	171 [167-173]	2744 [2652-2838]	470 [462-477]	3214 [3118-3311]
South Carolina	2193 [2103-2279]	141 [138-144]	203 [199-206]	2537 [2443-2624]	428 [421-435]	2965 [2865-3056]
Alabama	1998 [1911-2084]	192 [189-196]	233 [229-237]	2423 [2332-2512]	408 [401-414]	2831 [2737-2921]
Louisiana	2141 [2043-2222]	147 [144-150]	186 [181-189]	2474 [2370-2558]	344 [337-349]	2818 [2710-2904]
Maryland	2043 [1965-2132]	60 [59-62]	149 [145-151]	2252 [2172-2342]	448 [441-457]	2700 [2617-2795]
Indiana	2062 [1959-2136]	131 [127-132]	161 [157-163]	2354 [2246-2426]	345 [340-350]	2699 [2588-2773]
Missouri	1776 [1692-1845]	117 [116-121]	156 [152-158]	2049 [1964-2119]	311 [307-317]	2360 [2274-2433]
Colorado	1971 [1882-2064]	48 [45-49]	81 [79-84]	2100 [2009-2195]	248 [243-254]	2348 [2254-2444]
Colorado	1752 [1674-1840]	141 [139-145]	131 [129-134]	2024 [1943-2116]	246 [242-252]	2270 [2186-2365]
North Carolina	1595 [1533-1676]	110 [107-112]	150 [146-152]	1855 [1790-1936]	326 [320-332]	2181 [2114-2264]
Mississippi	1474 [1421-1558]	117 [115-120]	182 [178-186]	1773 [1717-1859]	273 [269-282]	2046 [1990-2136]
New Mexico	1407 [1339-1490]	63 [61-65]	93 [90-96]	1563 [1493-1647]	185 [180-190]	1748 [1676-1834]
Nevada	1369 [1294-1447]	34 [33-37]	82 [79-84]	1485 [1409-1564]	228 [222-233]	1713 [1633-1792]
Arkansas	1252 [1185-1320]	102 [100-105]	122 [118-124]	1476 [1408-1546]	184 [180-188]	1660 [1590-1734]
Washington	1323 [1264-1395]	40 [39-40]	65 [63-66]	1428 [1368-1499]	219 [214-224]	1647 [1585-1719]
Oklahoma	1107 [1036-1169]	111 [108-114]	120 [120-126]	1338 [1268-1404]	224 [219-230]	1562 [1492-1631]
Massachusetts	1056 [1001-1111]	41 [39-42]	87 [84-88]	1184 [1126-1238]	372 [366-378]	1556 [1494-1610]
Minnesota	1278 [1209-1346]	34 [33-35]	46 [46-50]	1358 [1290-1429]	161 [157-165]	1519 [1450-1590]
Wisconsin	990 [959-1065]	43 [42-47]	65 [62-66]	1098 [1065-1174]	182 [180-188]	1280 [1248-1358]
Connecticut	972 [933-1049]	31 [30-32]	60 [57-61]	1063 [1022-1140]	205 [204-213]	1268 [1228-1351]
Kansas	854 [800-923]	35 [34-38]	51 [49-54]	940 [886-1010]	112 [110-117]	1052 [997-1126]
Oregon	814 [758-863]	18 [17-19]	36 [36-39]	868 [814-919]	124 [120-128]	992 [937-1043]
Iowa	648 [591-690]	32 [30-34]	38 [37-40]	718 [660-761]	106 [102-109]	824 [764-868]
Utah	613 [561-670]	11 [11-13]	28 [28-31]	652 [601-711]	104 [100-108]	756 [704-818]
South Dakota	465 [424-515]	26 [23-27]	32 [29-34]	523 [479-573]	47 [44-49]	570 [524-621]
Idaho	457 [419-504]	16 [15-16]	24 [23-26]	497 [459-544]	71 [68-74]	568 [529-616]
Nebraska	447 [404-484]	17 [14-17]	21 [20-22]	485 [442-521]	60 [59-64]	545 [502-584]
West Virginia	375 [340-413]	41 [38-43]	44 [41-45]	460 [423-497]	75 [72-78]	535 [497-572]
Montana	317 [294-353]	6 [5-6]	17 [16-18]	340 [316-375]	43 [41-46]	383 [359-420]
Maine	332 [312-391]	8 [7-8]	8 [8-9]	348 [328-407]	34 [32-36]	382 [361-442]
North Dakota	293 [284-359]	14 [13-16]	17 [16-19]	324 [315-392]	27 [26-30]	351 [342-420]
Delaware	224 [203-255]	18 [17-19]	30 [28-31]	272 [249-304]	72 [71-77]	344 [323-379]
Rhode Island	168 [146-192]	12 [9-12]	22 [21-23]	202 [179-225]	71 [69-75]	273 [249-298]
Wyoming	183 [153-201]	13 [11-15]	13 [12-16]	209 [180-230]	23 [20-24]	232 [202-252]
New Hampshire	140 [108-148]	8 [7-8]	13 [11-14]	161 [128-170]	49 [47-52]	210 [177-219]
Hawaii	147 [119-163]	2 [2-2]	8 [7-9]	157 [128-174]	46 [43-51]	203 [174-222]
Alaska	156 [142-202]	3 [3-5]	11 [9-13]	170 [155-218]	21 [20-26]	191 [176-242]
Vermont	165 [140-190]	3 [2-3]	3 [2-3]	171 [146-196]	15 [14-17]	186 [161-211]
Total	108384 [107906-109132]	4516 [4504-4536]	7730 [7705-7750]	120630 [120145-121390]	22007 [21969-22085]	142637 [142151-143482]

References

[1] Hillis, S. D. *et al.* Global minimum estimates of children affected by covid-19-associated orphanhood and deaths of caregivers: a modelling study. *The Lancet* (2021).

[2] Lotka, A. *Théorie Analytique des Associations Biologique* (Hermann et Cie, 1939).

[3] CDC. Quarterly Excess Deaths by State, Sex, Age, and Race (2021). URL `https://data.cdc.gov/NCHS/AH-Quarterly-Excess-Deaths-by-State-Sex-Age-and-Ra/jqg8-ycmh`.

[4] Division of Vital Statistics, National Center for Health Statistics (NCHS), Centers for Disease Control and Prevention (CDC), United States Department of Health and Human Services (US DHHS) . Live births in the United States, 1995-2019, Natality public-use data on CDC WONDER Online Database for years 1995-2019 (2021). URL `https://wonder.cdc.gov/wonder/help/Natality.html#Source`.

[5] Division of Vital Statistics, National Center for Health Statistics (NCHS), Centers for Disease Control and Prevention (CDC), United States Department of Health and Human Services (US DHHS) . Live births in the United States, 2016-2019, Natality public-use data on CDC WONDER Online Database, for years 2016-2019 (expanded) (2021). URL `https://wonder.cdc.gov/wonder/help/Natality.html#Source`.

[6] U.S. Census Bureau. Single-Race Resident Population Estimates United States, State and County for the years 2010 - 2019 (2021). URL `https://wonder.cdc.gov/single-race-population.html`.

[7] U.S. Census Bureau. Table S1002, 2015-2019 American Community Survey 5-Year Estimates (2021). URL `https://data.census.gov/cedsci/table?tid=ACSST5Y2019.S1002`.

[8] United Nations Population Division. World Population Prospects - Population Division - United Nations (2020). URL `https://population.un.org/wpp/Download/Standard/Mortality/`.

[9] Bernal, J. L. *et al.* Transmission dynamics of COVID-19 in household and community settings in the United Kingdom. *medRxiv* (2020).

[10] Brazeau, N. *et al.* Report 34: COVID-19 infection fatality ratio: estimates from seroprevalence (2020). URL `https://doi.org/10.25561/83545`.
